# Membrane lipid composition of bronchial epithelial cells influences antiviral responses during rhinovirus infection

**DOI:** 10.1080/21688370.2023.2300580

**Published:** 2024-01-05

**Authors:** Madhuriben H. Panchal, Emily J. Swindle, Theresa J. Pell, Wendy C. Rowan, Caroline E. Childs, James Thompson, Benjamin L. Nicholas, Ratko Djukanovic, Victoria M. Goss, Anthony D. Postle, Donna E. Davies, Cornelia Blume

**Affiliations:** a Faculty of Medicine, School of Clinical and Experimental Sciences, University of Southampton, Southampton, UK; b Southampton NIHR Biomedical Research Centre, University Hospital Southampton NHS Foundation Trust and University of Southampton, Southampton, UK; c Institute for Life Sciences, University of Southampton, Southampton, UK; d GSK R&D, Stevenage, Hertfordshire, UK; e Faculty of Medicine, School of Human Development and Health, University of Southampton, Southampton, UK; f Biomedical Imaging Unit, Faculty of Medicine, University of Southampton, Southampton, UK

**Keywords:** Bronchial epithelium, membrane lipids, arachidonic acid, rhinovirus, severe asthma

## Abstract

Lipids and their mediators have important regulatory functions in many cellular processes, including the innate antiviral response. The aim of this study was to compare the lipid membrane composition of in vitro differentiated primary bronchial epithelial cells (PBECs) with ex vivo bronchial brushings and to establish whether any changes in the lipid membrane composition affect antiviral defense of cells from donors without and with severe asthma. Using mass spectrometry, we showed that the lipid membrane of in vitro differentiated PBECs was deprived of polyunsaturated fatty acids (PUFAs) compared to ex vivo bronchial brushings. Supplementation of the culture medium with arachidonic acid (AA) increased the PUFA-content to more closely match the ex vivo membrane profile. Rhinovirus (RV16) infection of AA-supplemented cultures from healthy donors resulted in significantly reduced viral replication while release of inflammatory mediators and prostaglandin E2 (PGE_2_) was significantly increased. Indomethacin, an inhibitor of prostaglandin-endoperoxide synthases, suppressed RV16-induced PGE_2_ release and significantly reduced CXCL-8/IL-8 release from AA-supplemented cultures indicating a link between PGE_2_ and CXCL8/IL-8 release. In contrast, in AA-supplemented cultures from severe asthmatic donors, viral replication was enhanced whereas *PTGS2* expression and PGE_2_ release were unchanged and CXCL8/IL-8 was significantly reduced in response to RV16 infection. While the PTGS2/COX-2 pathway is initially pro-inflammatory, its downstream products can promote symptom resolution. Thus, reduced PGE_2_ release during an RV-induced severe asthma exacerbation may lead to prolonged symptoms and slower recovery. Our data highlight the importance of reflecting the in vivo lipid profile in in vitro cell cultures for mechanistic studies.

## Introduction

The respiratory epithelium forms a physical, chemical and immunological barrier against inhaled environmental impacts, including pollen, particulates, bacteria and viruses. As the respiratory epithelium is the first site of contact for inhaled substances, the innate responses toward these agents influence the homeostasis of the airway barrier. It is thought that many chronic respiratory diseases, including asthma and COPD, are initiated by altered epithelial barrier responses.^1,2^

Asthma is a disease characterized by episodes of variable airflow obstruction that are triggered spontaneously or in response to environmental exposures. While these symptoms can be controlled or relieved by current therapies, people with asthma continue to experience exacerbations of their disease which contribute to disease morbidity across all ages. Up to 80% of asthma exacerbations in children are associated with viral infections with rhinovirus infections the most prevalent virus class.^3,4^

Rhinoviruses possess a small (ca. 7.5 kb) single positive-strand RNA genome and are members of the picornavirus family. Following infection, respiratory epithelial cells mount an innate response, including the induction of Type I and Type III interferons (IFNs).^5^ These IFNs launch an immediate, intense local response within an infected cell, triggering expression of IFN-stimulated genes (ISGs) that contribute to anti-viral defense mechanisms or, if necessary, elicit apoptosis of the infected cell to limit viral replication. IFNs also act locally by inducing an anti-viral state in uninfected cells and signal to recruit immune cells to the site of infection.^6,7^ While many protein products of ISGs contribute to the coordination of the anti-viral defense mechanisms,^8^ lipid mediators derived from polyunsaturated fatty acids (PUFAs) in the lipid membrane, especially arachidonic acid (AA or 20:4n-6) as precursor of eicosanoids, e.g. prostaglandin E2 (PGE_2_), are also important signaling molecules involved in cellular signaling events, e.g. during infection and inflammation.^9^

Human *in vitro* cell culture models, including cell lines and primary cells, are commonly used in respiratory research for mechanistic studies to analyze cellular responses to environmental stimuli such as rhinovirus and characterize the signaling pathways involved.^10,11^ These models have been used to compare responses of cells from control subjects with those from severe asthmatic donors and have identified differences in viral replication and release of type I and III IFNs that contribute to asthma exacerbations.^12,13^ While the importance of the link between cell function and the lipid membrane composition has been known for more than 30 years,^14^ this aspect is still largely overlooked when using human *in vitro* cell cultures. The composition of the lipid membrane in cell cultures is dependent on the fatty acid content of the serum supplement used,^15^ yet in most cases animal derived serum or extracts (e.g. bovine pituitary extract) are employed as the main source of lipids. Consequently, the lipid profile of cultured cells can be markedly different compared to cells *in vivo*.^16^ However, the functional consequences of such an altered lipid profile of *in vitro* cultures are widely neglected. Consequently, in many human *in vitro* cell culture experiments signaling via lipid mediators may not appropriately reflect *in vivo* responses.

It is well accepted that cytosolic phospholipase A2 (cPLA_2_ encoded by *PLA2G4A*) plays a central role in mobilizing AA from the pool of glycerophospholipids for eicosanoid biosynthesis upon cell activation.^17^ Downstream in prostanoid biosynthesis, AA is oxidized involving prostaglandin endoperoxide synthases (PTGS1 and PTGS2, also referred to as cyclooxygenase 1 (COX1) and cyclooxygenase 2 (COX2)) to prostaglandin H2 (PGH_2_) which is a central intermediate in prostaglandin synthesis. PGH_2_ can be further processed into PGE_2_ by prostaglandin E synthase (PTGES).^18,19^ The release of PGE_2_ by respiratory epithelial cells has been shown to be induced during viral infections,^20–22^ and PGE_2_ has also been shown to modulate immune cell function^23^ as well as to modify the IFN response during viral infections.^24,25^

In this study, we hypothesized that the membrane lipid profile plays an important role in the innate immune responses to rhinovirus infection in severe asthma. Therefore, our aim was to compare the lipid membrane profile of *ex vivo* human bronchial brushings with *in vitro* differentiated PBECs and adjust the *in vitro* lipid membrane profile to resemble better the *ex vivo* profile by supplementing the culture medium with selected PUFAs. We identified that when the *in vitro* lipid membrane more closely mirrored the *ex vivo* profile, differentiated PBECs derived from non-asthmatic donors were more resistant to rhinovirus infection as shown by a decrease in viral replication. This was associated with an increase in expression of *PTGS2*, release of PGE_2_ and inflammatory mediators. The importance of PGE_2_ in cultures from non-asthmatic donors was demonstrated by treatment with indomethacin which inhibited PGE_2_ release and reversed the PUFA-induced increase in IL-8 release during viral infection. In contrast, differentiated PBECs derived from donors with severe asthmatic showed an enhancement of viral replication while expression of *PTGS2* and release of PGE_2_ were not increased. Interestingly, virally induced release of IL-8 was reduced by PUFA supplementation.

## Methods

### Subjects and primary cell culture

Human PBECs were obtained by brush biopsies using fiberoptic bronchoscopy from subjects without asthma and with severe asthma recruited from a volunteer database (Supplementary Table S1). Subjects with severe asthma were classified according to the BTS/SIGN guidelines as step 4 of asthma management. All volunteers in the group with severe asthma received regular treatment with high-dose inhaled corticosteroids (ICS) and long acting beta agonists (LABA). All procedures were approved by the Southampton and South West Hampshire Research Ethics Committee (05/q1702/165 and 09/H0504/109) and were undertaken following informed consent. Bronchial brush biopsies were placed in sterile PBS and an aliquot of these *ex vivo* cells stored at −80°C for lipid analysis. The remaining cells were cultured in bronchial epithelial growth medium (Lonza, Basel, Switzerland) and differentiation was induced at passage 2 as previously described.^26^ Briefly, PBECs were plated on Transwell permeable supports (diameter 6.5 mm, polyester membrane with 0.4 µm pores, Corning Life Sciences, Amsterdam, The Netherlands) and differentiated at an air-liquid interface (ALI) for 21 days. Transepithelial electrical resistance (TER) was monitored weekly using a EVOM voltohmmeter (World Precision Instruments, Aston, UK) and cells with a TER > 330 Ω.cm^2^ on day 21 were used for experiments. After this period of differentiation, the cell culture medium was supplemented with 30 µM linoleic acid (LA or 18:2n-6), arachidonic acid (AA or 20:4n-6) or docosahexaenoic acid (DHA or 22:6n-3) adsorbed to fatty acid free BSA (1 mg/ml final concentration, PAA Laboratories, Pasching, Austria) on days 1, 3 and 5; control cultures were supplemented with BSA alone. Cultures were harvested on day 6. Differentiation status was assessed by TER measurements and % ciliation by high speed video microscopy as previously described.^27^

### Rhinovirus infection

Human rhinovirus (RV16; ATCC VR-283™, Teddington, UK) was amplified using H1 HeLa cells as previously described.^28^ Differentiated PBECs were supplemented with PUFAs on day 1 and 3 before HRV16 infection on day 5, as described above. After measuring TER on day 5, basolateral medium was changed (± PUFA supplementation as indicated) and cells were infected with human RV16 at a multiplicity of infection (MOI) of 1 for 6 h; after infection residual virions were removed by apical washing (3×) using HBSS and then the cultures were incubated for an additional 18 h (infection time 24 h in total) at the air-liquid interface. Ultraviolet-irradiated virus controls (UV-RV16) were prepared by exposure of virus stocks to UV light at 1200 mJ/cm^2^ on ice for 50 min. In some experiments, 1 µM indomethacin (Sigma) was added to fresh basolateral medium after the TER measurements on day 5 of supplementation and cultures were infected with RV16 after a preincubation period of 1 h with indomethacin. TER was measured after 24 h of rhinovirus infection and apical washes were collected and basolateral supernatants harvested, centrifuged to remove cell debris and stored at −20°C until further analysis. Cells on permeable supports were frozen at −80°C for lipid extraction or used for RNA analysis. Release of infective virions in apical cell culture washes was determined using a HeLa titration assay and 50% tissue culture infective dose assay (TCID50/ml).

### Analysis of lipid membrane profile

Lipids from *ex vivo* bronchial brushes and *in vitro* differentiated PBECs were extracted using the Bligh and Dyer method. After thawing at room temperature, 800 µl of 0.9% saline solution and 50 µl of an internal standard mixture were added to each sample to enable phospholipid quantification using synthetic lipid standards (see Supplementary Table S2). After adding 10 µl of butylated-hydroxy-toluene (BHT) solution, each sample was vigorously mixed using vortex and lipids extracted using a Freedom EVO 100 robotic liquid handling system (Tecan, Theale UK), with 24 samples being extracted in each batch. The program was set to add 2 ml of methanol, 2 ml of dichloromethane (DCM) and 1 ml of distilled water to each sample followed by a mixing step to allow biphasic formation of a lower DCM layer containing phospholipid and an aqueous upper layer. The mixture was centrifuged at 3000rpm, 20°C for 10 minutes. The robot was programmed to remove the lipid layer which was then dried under a stream of warmed Nitrogen gas using an Ultravap (Porvair, Norfolk, UK) for 40 minutes. Once the samples were dried, they were stored at −20°C until they were analyzed by mass spectrometry.

Mass spectrometric analysis was performed on a Waters XEVO TQ-MS instrument using electrospray ionization (ESI). Dried samples were dissolved in 350 µl 66% methanol, 30% DCM, 3% H_2_0 and 1% concentrated ammonia solution (NH_4_OH). Samples were injected by direct infusion. Phosphatidylcholine (PC) species were preferentially detected using positive ionization, whereas phosphatidylethanolamine (PE) and acidic phospholipids were quantified under positive ionization conditions. After fragmentation with argon gas, PC molecules produced a fragment with m/z + 184 corresponding to the protonated phosphocholine head group, and parent scans of the m/z 184 moiety provided diagnostic determination of PC. Phosphatidylglycerol (PG) and phosphatidic acid (PA) species were detected by precursor ion scans that generated a common glycerophosphate fragment of m/z −153, phosphatidylserine (PS) species by neutral loss scans of serine (m/z −87), and phosphatidylinositol (PI) species by precursor ion scans of the common dehydrated inositol phosphate fragment with m/z −241. Molecular lipid species and their corresponding mass used to identify phospholipids are shown in Supplementary Table S3. Spectra were processed using a visual basic macro program developed in-house. Initially, MS spectra were smoothed, baseline subtracted, and exported to individual Excel files. These files were then imported into the macro program and corrected for ^12^C or ^13^C isotopic effects. The phospholipid species were expressed as percentages of their respective totals present in the sample.

### Detection of mediator release (PGE_2_, IL-8, IP-10 and IL-29/28)

After 24 h of RV16 infection, released mediators in basolateral supernatants were detected using ELISA. PGE_2_ was detected using a Prostaglandin E2 ELISA Kit – monoclonal (Cayman Chemical) and IL-8, IP-10 and IL29/28 were measured using DuoSet ELISA Development Systems (R&D systems) according to the manufacturers’ instructions.

### Analysis of RNA expression levels by RT-qPCR

RNA was isolated from cell cultures 24 h after RV16 infection using standard phenol-chloroform extraction, and reverse transcribed to cDNA using a Precision Reverse Transcription kit (PrimerDesign, Southampton, UK) according to the manufacturer’s instructions. Using qPCR (cycling conditions 95°C 10 min, then 50 cycles of 95°C 15 s, 60°C 1 min) and primers listed in Supplementary Table S4 relative expression levels were determined using the ΔΔCt method. Data were normalized to the geometric mean of the housekeeping genes of each sample (ubiquitin C and glyceraldehyde 3-phosphate dehydrogenase) and fold change in gene expression was determined relative to the mean CT value of standard, uninfected non-asthmatic controls.

### Statistical analysis

Statistical evaluation was performed using the software GraphPad Prism 9.3.1. If not stated otherwise, related samples were analyzed for statistical significance using the non-parametric Wilcoxon test and unrelated samples using the non-parametric Mann-Whitney test. Differences were regarded as significant when *P* ≤ 0.05.

## Results

### Lipid profile of *ex vivo* bronchial brushings versus *in* *vitro* differentiated PBEC cultures

**Figure 1.**
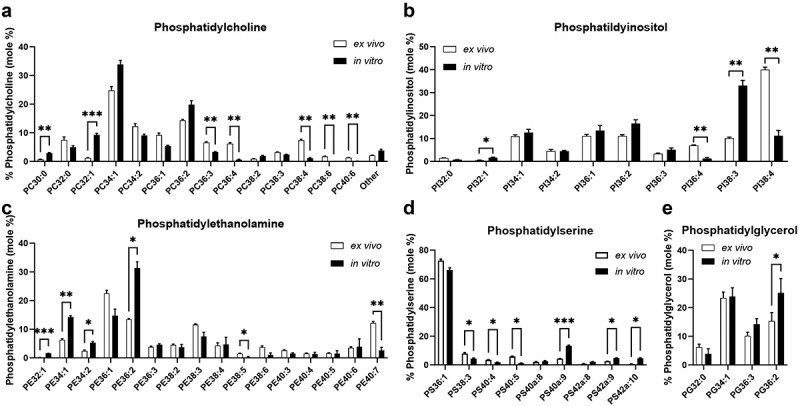
The lipid membrane profile of *in vitro* human differentiated PBECs is changed during culture compared to *ex vivo*. Cells from five different individuals were obtained by bronchial brush biopsy. Subsequently, one aliquot of cells was directly taken for analysis of the lipid profile by mass spectrometry (*ex vivo*) and the remaining cells were cultured *in vitro* and differentiated at the air-liquid interface as described in the methods section. The lipid profile of fully differentiated *in vitro* cultures of human PBECs was analyzed by mass spectrometry to generate matching pairs of *ex vivo* and *in vitro* cells. MEAN±SEM; *n* = 5 matching samples of individual subject. *: *p* ≤ 0.05, **: *p* ≤ 0.01, ***: *p* ≤ 0.001 2-way ANOVA with Bonferroni multiple comparison test.

To determine if the membrane lipid profiles changed during *in vitro* culture, the composition of the membrane lipids was analyzed by mass spectrometry. Initial comparisons were made between *ex vivo* bronchial epithelial brushings from five healthy, non-asthmatic subjects and their corresponding *in vitro* differentiated bronchial epithelial cultures. Here we determined the glycerophospholipid composition, which are classed into groups according to their head group (Supplementary Fig. S1). The fractional concentrations of phosphatidylcholine (PC), phosphatidylinositol (PI), phosphatidylethanolamine (PE), phosphatidylserine (PS) and phosphatidylglycerol (PG) are shown in Figure 1 and Supplementary Table S5. Representative mass spectra for PCs and PIs are shown in Supplementary Fig. S2 and S3. The variability between different individuals was relatively small within the *ex vivo* or *in vitro* groups. However, we observed significant differences in the lipid profile between *ex vivo* and *in vitro* cultured cells. *Ex vivo* bronchial brushings showed higher amounts of phospholipids containing polyunsaturated fatty acids (PUFAs), including the arachidonate-containing phospholipids PC36:4 (PC16:0/20:4) and PC38:4 (PC18:0/20:4) (Figure 1a), PI36:4 (PI16:0/20:4) and PI38:4 (PI18:0/20:4) (Figure 1b). Fractional concentrations of all these phospholipid species were significantly lower in differentiated cells *in vitro*. This change was particularly apparent for PI38:4 (PI18:0/20:4), the % concentration of which decreased significantly from 40.0 ± 1.08% *ex vivo* to 11.2 ± 2.33% *in vitro* in cells from healthy non-asthmatic subjects (Supplementary Table S5). No significant differences were observed for PS species, while for PGs only PG36:3 (PG18:1/18:2) showed a significant increase in *in vitro* cultured cells compared to *ex vivo* brushings (Figure 1(d,e) Supplementary Table S5). Phospholipid species measured by shotgun lipidomics by mass: charge frequently contain a number of isobaric individuals with different combinations of fatty acyl groups. Consequently, a number of ions were selected in negative ionization for MS/MS product scans of constituent fatty acid groups. While, for instance, in *ex vivo* bronchial brushing samples PI38:4 contains over 85% of the arachidonoyl species PI18:0/20:4, this species comprises only some 40% PI38:4 *in vitro* differentiated PBECs using standard culture media with the remaining 60% being PI18:1/20:3. This substitution was similar for all other putative arachidonoyl-containing species of PC, PI and PE, indicating a severe deficit of arachidonoyl species *in vitro*. The compositions of all these lipid classes *in vitro* was dominated by species containing unsaturated fatty acids that could be synthesized *de novo* by fatty acid synthase, predominantly oleate (18:1) and trieicosanoate (Mead acid, 20:3). Mead acid is a marker of essential fatty acid deficiency^29^ and was predominantly located in the PI fraction as PI38:3 (PI18:0/20:3) for cells *in vitro*, in an apparent abortive attempt to substitute the essential signaling role of PI18:0/20:4. Similar fatty acid product scans demonstrated that the phospholipid species with increased concentrations *in vitro* (PC32:1, PC36:1, PC38:2, PE36:1, PE38:2) were dominated by oleoyl-containing individual species. For instance, PC38:2 and PE38:2 are predominately 18:0/18:2 *ex vivo* but essentially 18:1/18:1 *in vitro*.

Interestingly, the medium supplement used for culturing PBECs *in vitro*, bovine pituitary extract, had a relative high content of saturated and mono-unsaturated fatty acids while the content of polyunsaturated fatty acids was low (Table 1). For example, the content of the saturated palmitic acid (16:0) and stearic acid (18:0) and the mono-unsaturated oleic acid (18:1n-9) was 563.2 ng/ml, 439.2 ng/ml and 455.2 ng/ml in the culture medium, respectively, while the concentration of poly-unsaturated arachidonic acid (20:4n-6) and linoleic acid (18:2n-6) was 93.6 ng/ml and 162.4 ng/ml, respectively. Taken together, our data show that during *in vitro* culture with an animal derived supplement that is relatively low in polyunsaturated but high in mono-unsaturated and unsaturated fatty acids, the lipid profile of PBECs is changed toward a lower content of PUFAs, especially arachidonic acid containing glycerophospholipids.

**Table 1.** Fatty acid content of the medium supplement. Bovine pituitary extract (BPE) was used as a lipid containing medium supplement for *in vitro* cultured and differentiated PBECs at a dilution of 1:250.

Fatty acid	% of total fatty acid content	µg/mL BPE (medium supplement)	ng/mL in culture medium
14:0	10.5	71.6	286.4
15:0	4.2	28.8	115.2
16:0	20.7	140.8	563.2
16:1n-7	0.8	5.2	20.8
17:0	1.0	6.8	27.2
18:0	16.1	109.8	439.2
18:1n-9	16.7	113.8	455.2
18:1n-7	1.2	8.0	32.0
18:2n-6	6.0	40.6	162.4
18:3n-6	0.5	3.2	12.8
18:3n-3	6.1	41.6	166.4
20:0	0.2	1.4	5.6
20:1n-9	3.8	25.6	102.4
20:2n-6	6.2	42.4	169.6
20:3n-6	0.4	3.0	12.0
20:4n-6	3.4	23.4	93.6
20:4n-3	0.1	0.8	3.2
20:5n-3	2.0	13.8	55.2
TOTAL	100.0	680.6	2722.4

### PUFA supplementation of *in* *vitro* differentiated PBEC cultures moves the lipid profile closer to that observed in *ex vivo* bronchial brushings

**Figure 2.**
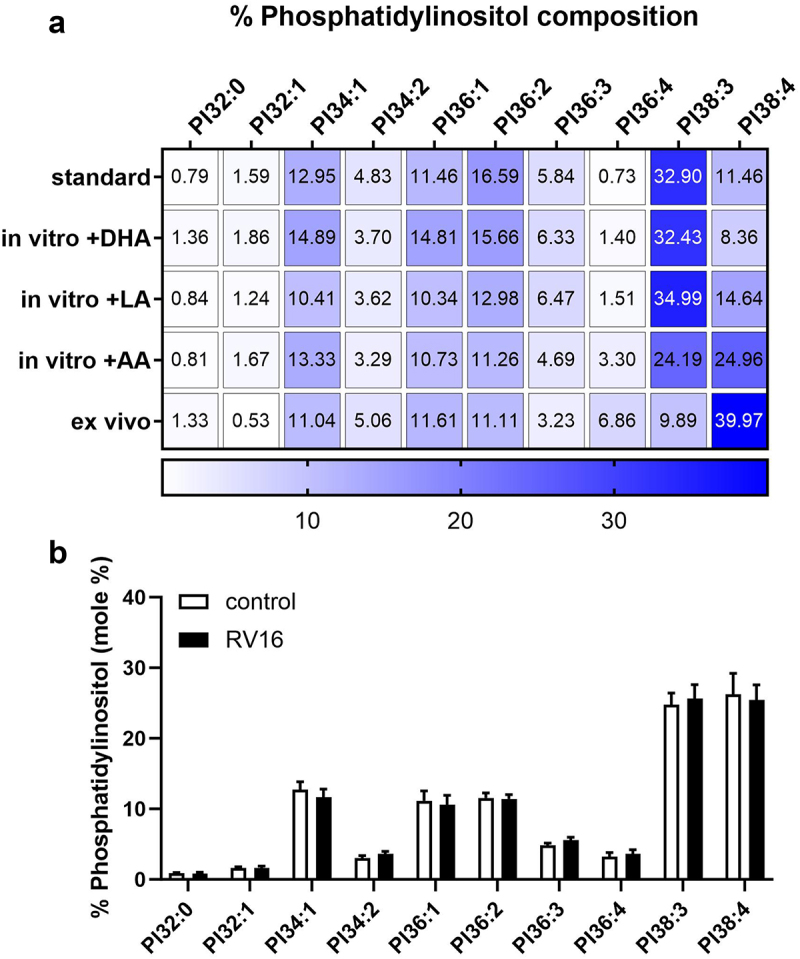
PUFA supplementation of *in vitro* differentiated human PBEC cultures altered the lipid membrane profile similar to *ex vivo*. Differentiated PBEC cultures were supplemented with lipids as specified in the methods section and the lipid membrane profile analyzed using mass spectrometry. a: comparison of the phosphatidylinositol (PI) profile standard culture conditions (*n* = 5) and supplemented with arachidonic acid (AA) (*n* = 5), linoleic acid (LA) (*n* = 4) or docosahexaenoic acid (DHA) (*n* = 3). For comparison, the PI profile of *ex vivo* bronchial brushings are shown (*n* = 5). MEAN; matching samples of individual subject. b: PI composition of *in vitro* differentiated PBECs of healthy subjects supplemented with AA after 24 h of RV16 infection compared to control uninfected AA-supplemented cultures (*n* = 5).

Since the contents of PUFAs, especially arachidonic acid (AA), were reduced in phospholipids from differentiated PBECs *in vitro*, we supplemented the culture medium of differentiated PBECs with PUFAs complexed to fat free albumin for five days and compared their lipid membrane profiles to control (i.e. standard, unsupplemented) cultures and *ex vivo* bronchial brushings (Supplementary Table S6). Since PIs have been shown to play an important role in regulating cellular processes,^30^ their composition following PUFA supplementation is shown in Figure 2(a). Supplementation of *in vitro* cultures with PUFAs partially restored lipid profiles toward those observed *ex vivo*. In particular, supplementation with AA resulted in a significant increase in PC32:0 (PC16:0/16:0), PC38:4 (PC18:0/20:4), PC40:6 (PC18:0/22:6), PI38:4 (PI18:0/20:4) and PS40:4 (PS18:0/22:4) and a significant decrease in PS40a:9 (PS20:5a/20:4) which is closer to the *ex vivo* profile. Compared to control *in vitro* cultures, LA supplementation significantly increased the content of PC32:0 (PC16:0/16:0), PC36:3 (PC18:1/18:2), PC38:4 (PC18:0/20:4) and PG32:0 (PG16:0/16:0) and a significant decrease in PS40a:9 (PS20:5a/20:4) closer to the levels found *ex vivo*. In contrast, DHA supplementation did not cause any significant changes in the lipid composition. In summary, supplementation of the standard culture medium with AA and LA increased the content of PUFAs in glycerophospholipids in *in vitro* differentiated bronchial epithelial cultures, counteracting the deprivation of PUFAs caused by *in vitro* culture using animal derived supplements. Supplementation with AA increased the content of PUFAs in the lipid membranes, especially the ratio of PI38:4 (PI18:0/20:4) to PI38:3 (PI18:0/20:3), to be more closely representative of the *ex vivo* profile. Of note, we did not observe any changes in the differentiation status of bronchial epithelial cultures following AA supplementation as the Transepithelial Electrical Resistance (TER) as well as the surface area covered by moving cilia determined by high speed video microscopy was not significantly changed following AA supplementation (Supplementary Fig. S4A-D). Additionally, the expression of *ICAM-1*, which facilitates RV16 virus entry into cells, as well as the expression of *TLR3* and *IFIH1* supporting detection of RV16 infection in cells,^31^ were not significantly changed following AA supplementation (Supplementary Fig. S4E-G).

### AA supplementation alters the antiviral response in *in* *vitro* differentiated PBEC cultures

**Figure 3.**
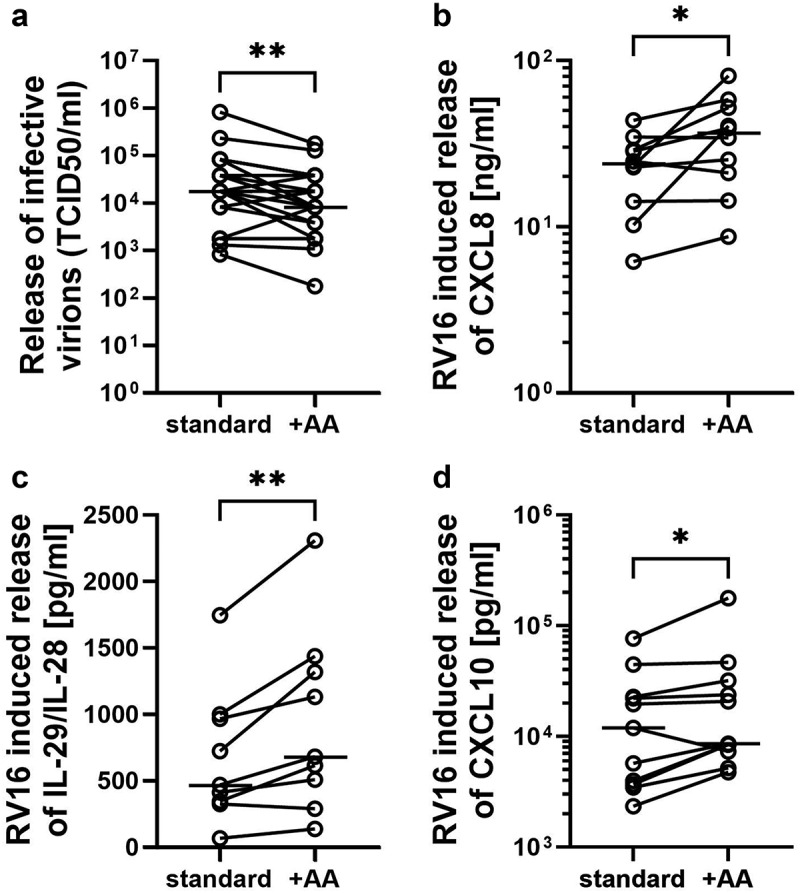
AA supplementation alters the response of *in vitro* differentiated human PBECs during rhinovirus infection. Differentiated *in vitro* cultures of human PBECs were supplemented with AA as described in the method section. Standard un-supplemented cultures were used as control. Cultures were infected with human rhinovirus 16 (RV16) for 24hrs before supernatants were taken. Viral replication was determined by the release of infective virions using TCID50 assays (a). Release of CXCL8 (IL-8) (b), IFNλ (IL29/IL28) (c) and CXCL10 (IP-10) (d) was analyzed by ELISA. A: *n* = 21; B: *n* = 10; C: *n* = 9; D: *n* = 11; *: *p* ≤ 0.05, **: *p* ≤ 0.01 non-parametric Wilcoxon test.

**Figure 4.**
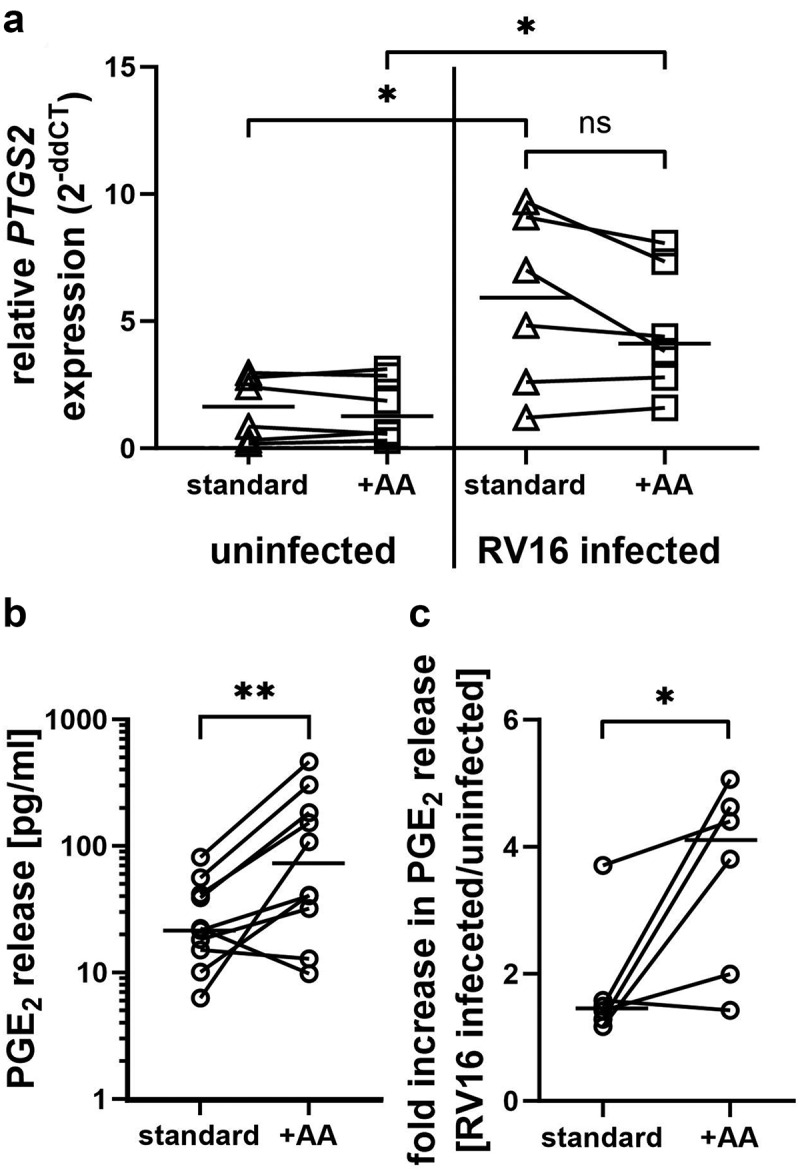
Increased PGE_2_ release during RV16 infection in AA supplemented cultures. Differentiated human PBECs derived from non-asthmatic subjects were supplemented with or without AA and infected with rhinovirus 16 (RV16) for 24hrs. a: expression of *PTGS2* was determined by qPCR. *n* = 6. b and c: PGE_2_ release into the basolateral medium over a period of 24 h was determined by ELISA. b: PGE_2_ release of uninfected cultures with and without AA supplementation. *n* = 9. c: RV16-induced PGE_2_ release was normalized to the respective uninfected control. *n* = 6. *: *p* ≤ 0.05, **: *p* ≤ 0.01, non-parametric Wilcoxon test for paired samples, Mann-Whitney test for unpaired samples.

In order to test if the observed changes in the lipid membrane profile had an impact on the innate immune responses of the airway epithelium, we next compared the antiviral responses of control and AA-supplemented differentiated PBECs from non-asthmatic subjects during human rhinovirus 16 (RV16) infection. Control experiments showed that the lipid membrane profile of AA supplemented *in vitro* cultures was maintained during rhinovirus infection (Figure 2b). Compared to standard *in vitro* differentiated cultures, release of infective virions as determined by TCID50 assay was significantly reduced following AA-supplementation (Figure 3a). (standard conditions: TCID50/ml of 17,582 (4960–59744); AA supplementation: TCID50/ml 8161 (2773–37880) (median (IQR)). In contrast, viral copy number was not significantly changed following AA supplementation (RV16 copy number: standard conditions: 22698 (16868–35494); AA supplementation: 29185 (7529–66869) (median (IQR)) suggesting that increased levels of AA might preferentially affect virion assembly rather than viral RNA replication. While release of infectious virus particles was decreased in AA supplemented cultures, there was a significant increase in release of IL-8 (standard cultures 23.9 (13.2–30.3) ng/ml, AA-supplementation 36.7 (19.3–53.6) ng/ml; median (IQR)). Release of IL-29/IL-28 and interferon-stimulated protein CXCL10 (IP-10) (Figure 3b–d) was also significantly increased in the same cultures infected with RV16 (IL-29/IL-28: standard cultures: 467.0 (334.3–982.9) pg/ml, AA-supplementation: 679.9 (397.9–1377) pg/ml; CXCL10: standard cultures: 11900 (3684–22700) pg/ml, AA-supplementation: 8599 (7296–31900) pg/ml; median (IQR)),

Since supplementation of *in vitro* differentiated PBECs with AA resulted in the highest increase of PI38:4, an important precursor in the generation of prostaglandins, we investigated the PGE_2_ production pathway during RV16 infection. As shown in Figure 4(a), the expression of *PTGS2* (COX-2) was unchanged during AA supplementation at baseline. However, *PTGS2* expression increased significantly during RV16 infection with no difference between un- and AA-supplemented cultures. The expression of other rate-limiting enzymes in the production of PGE_2_ was not increased by AA supplementation or RV16 infection in the cultures (Supplementary Fig. S5). There was a trend for RV16 infection to suppress *PTGS1* expression, although this only reached significance for standard cultures (Supplementary Fig. S5). Interestingly, release of PGE_2_ in uninfected control cultures with AA supplementation was significantly higher in basolateral culture supernatants of *in vitro* differentiated PBECs than in similarly treated unsupplemented control cultures (Figure 4b). In response to infection with RV16, release of PGE_2_ was increased, with AA supplemented cultures showing a significantly higher fold increase release from baseline levels than standard cultures (Figure 4c).

### Inhibition of PTGS reduces RV16 induced release of PGE2 and IL-8

**Figure 5.**
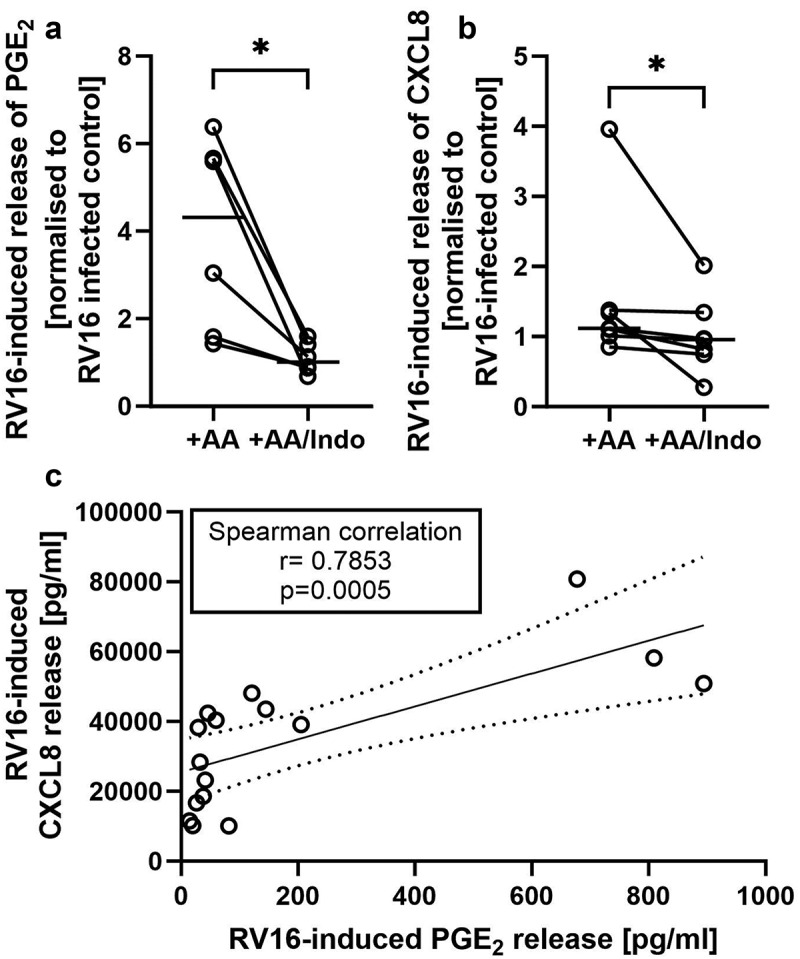
RV16-induced PGE2 and CXCL8 (IL-8) release is altered by PTGS inhibition. Standard un-supplemented and AA-supplemented *in vitro* cultures of differentiated human PBECs from non-asthmatic donors were infected with human rhinovirus 16 (RV16) for 24hrs. a and b: cultures were incubated with 1 μM indomethacin (indo) for 1 h prior RV16 infection in order to inhibit prostaglandin-endoperoxide synthase (PTGS). Basolateral release of PGE2 (a) and CXCL8 (b) was determined by ELISA. Results are normalized to the RV16 induced mediator release of the respective unsupplemented control. c: the release of PGE_2_ correlates with the CXCL8 release during RV16 infections. A: *n* = 6; B: *n* = 7; c: *n* = 16; *: *p* ≤ 0.05, non-parametric Wilcoxon test for paired samples.

**Figure 6.**
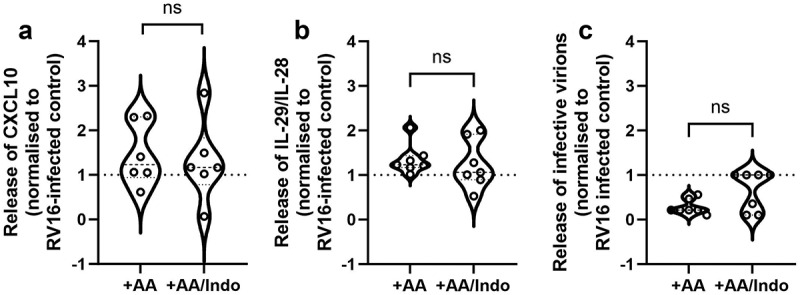
PTGS inhibition does not affect RV16 replication or RV16 induced release of IL-29/IL-28 and CXCL10 (IP-10) in AA supplemented cultures. Standard un-supplemented and AA supplemented *in vitro* cultures of differentiated human PBECs from non-asthmatic donors were infected with human rhinovirus 16 (RV16) for 24hrs. Cultures were incubated with 1 μM indomethacin (indo) for 1 h prior RV16 infection in order to inhibit prostaglandin-endoperoxide synthase (PTGS). Release of CXCL10 (a) and IL-29/IL-28 (b) into the basolateral supernatant was determined by ELISA. Results are normalized to the RV16-induced mediator release of the respective unsupplemented control. Release of infective virions into the apical wash was determined by TCID50 assay (c). a: *n* = 6; b: *n* = 7; c: *n* = 7; non-parametric Wilcoxon test for paired samples.

We next investigated the role of RV16 induced PGE_2_ release in the antiviral response by using the PTGS inhibitor indomethacin. As shown in Figure 5(a), indomethacin significantly reduced the RV16 induced release of PGE_2_ in AA supplemented cultures. Furthermore, the RV16-dependent release of IL-8 was significantly reduced by indomethacin in AA supplemented cultures (Figure 5b). Of note, the amount of PGE_2_ released during RV16 infection correlated significantly (Spearman correlation, *r* = 0.7853; *p* = 0.0005) with the amount of IL-8 release (Figure 5c). In contrast, there was no significant effect of indomethacin on RV16 induced release of CXCL10/IP-10 or IL-28/29 (Figure 6a,b). There was also a trend for indomethacin to reverse the suppressive effect of AA supplementation on viral replication, however, this effect was not statistically significant (Figure 6c). In summary, these data indicate that lipid supplementation influences viral replication, as well as antiviral and inflammatory responses of differentiated PBEC cultures from non-asthmatic donors.

### AA supplementation has a distinct effect on the antiviral responses of PBEC cultures derived from subjects with severe asthma

Since it is known that the anti-viral response of the respiratory epithelium is changed in asthma, we next investigated the effect of AA supplementation on the response to RV16 infection in *in vitro* differentiated PBECs derived from subjects with severe asthma. First, we compared the lipid membrane profiles of *ex vivo* bronchial brushings and *in vitro* differentiated cultures. We did not observe any significant differences in the lipid membrane profile of bronchial brushings derived from subjects with severe asthma compared to subjects without asthma (Supplementary Fig. S6A and Supplementary Tables S5 and S7). Furthermore, the alterations in the lipid profile of *ex vivo* compared to *in vitro* differentiated bronchial epithelial cells from donors with severe asthma was similar to those observed in cells from non-asthmatic subjects, with *in vitro* differentiated cultures of subjects with severe asthma showing a similar reduction in lipids containing arachidonoyl species (Supplementary Fig. S6B and Supplementary Tables S5 and S7). We did not observe any significant differences in the differentiation status of PBECs cultures derived from donors with severe asthma following supplementation with AA as determined by TER and % ciliation (Supplementary Fig. S7A-D). We also observed no differences in the expression of genes involved in viral infection (*ICAM-1, TLR-3, and IFIH1*) of the differentiated cultures following AA supplementation (Supplementary Fig. S7E-G). PUFA supplementation of cultures derived from donors with severe asthma resulted in changes in the lipid membrane composition which were similar to those observed in cultures from non-asthmatic donors (Supplementary Fig. S6C, Supplementary Tables S6 and S8).

**Figure 7.**
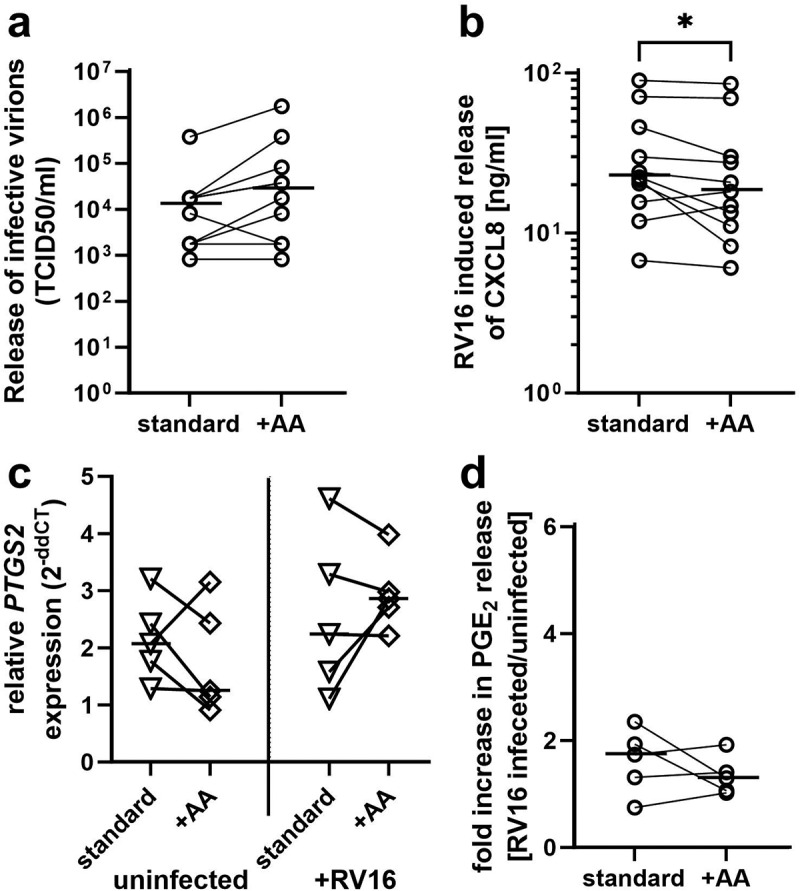
In PBECs derived from subjects with severe asthma lipid supplementation results in increased release of infective virions and reduced release of IL-8 during RV16 infection while PTGS2 expression and PGE_2_ release is unaffected. Standard un-supplemented and AA supplemented *in vitro* cultures of differentiated human PBECs derived from subjects with severe asthma were infected for 24 h with human rhinovirus 16 (RV16). a: release of infective virions was measured in the apical wash by TCID50 assay. *n* = 10. b: basolateral release of CXCL8 (IL-8) after RV16 infection was analyzed by ELISA. (*n* = 11). c: expression of PTGS2 was determined by qPCR following RV16 infection. *n* = 5. d: release of PGE_2_ after RV16 infection into basolateral supernatants was measured by ELISA. *n* = 5. *: *p* ≤ 0.05, non-parametric Wilcoxon test for paired samples.

In contrast to responses of AA-supplemented PBEC cultures from non-asthmatic donors, differentiated PBEC cultures derived from severe asthmatic donors showed a significant increase in viral replication following AA supplementation (Figure 7a). In standard cultures of differentiated PBECs derived from donors with severe asthma we observed a TCID50/ml of 12,872 (1758–17582) (median (IQR)), while AA supplementation significantly increased the release of infective virions to a TCID50/ml of 27,731 (1758–1555905) (median (IQR)). Similar to cultures derived from non-asthmatic donors, no significant difference was observed in the viral copy number after AA-supplementation following RV16 infection of differentiated PBECs derived from patients with severe asthma (RV16 copy number: standard conditions: 25868 (12349–86086); AA supplementation: 59143 (19361–68448) (median (IQR)). Additionally, cultures derived from donors with severe asthma showed a significant reduction in RV16-induced release of IL-8 following AA supplementation (standard cultures: 22.4 (15.7–46.1) ng/ml, AA-supplemented 18.2 (11.2–30.0) ng/ml; median (IQR)) (Figure 7b). In contrast to responses of AA-supplemented PBEC cultures from non-asthmatic donors, RV16 infection did not affect expression of *PTGS2* in cultures from severe asthmatic subjects and release of PGE_2_ was unchanged (Figure 7c,d). There was no significant difference in the level of *PTGS2* expression in uninfected cultures derived from non-asthmatic donors or patients with severe asthma. However, following AA-supplementation the RV16-induced fold release of PGE_2_ in cultures from patients with severe asthma (1.297 (1.040–1.665); median (IQR)) was significant lower compared to non-asthmatic controls (4.106 (1.856–4.738); median (IQR)). Indomethacin did not affect PGE_2_ release during RV16 infection in AA-supplemented PBEC cultures from severe asthmatic donors (data not shown). Furthermore, there was no significant correlation between PGE_2_ and IL-8 release during RV16 infection (Spearman correlation, *r* = 0.5035; *p* = 0.0989). While RV16-induced release of IL-29/IL-28 and CXCL10 (IP-10) was significantly increased following AA supplementation (IL-29/IL-28: standard cultures: 254.9 (105.2–374.8) pg/ml; AA-supplementation: 510.9 (224.8–636.7) pg/ml; CXCL10: standard cultures: 5136 (2163–13213) pg/ml; AA-supplementation: 10700 (3430–17898) pg/ml; median (IQR)) (Supplementary Fig. S8), it was noted that IL-29/IL-28 and CXCL10 release from these cultures was lower than from healthy donors as reported previously (Figure 3c,d). However, while we were able to compare the effect of AA supplementation in each donor group due to the use of matched donor pairs (±AA), our study is not powered to directly compare the response between non-asthmatic donors and subjects with severe asthma.

In summary, these data indicate that lipid supplementation differentially affects viral replication in respiratory epithelial cells depending on the disease status of the donor. This has implications for mechanistic studies, especially those relating to antiviral immunity and the involvement of lipid mediators.

## Discussion

In our study, we showed that the *ex vivo* lipid membrane profile of bronchial brushings from non-asthmatic and severe asthmatic donors is similar. However, once these cells are placed in culture and differentiated *in vitro*, the PBECs become deprived of PUFAs, especially arachidonic acid containing glycerophospholipids. We further demonstrated that by supplementing the culture medium with arachidonic acid, the lipid profile could be recovered to more closely reflect the *ex vivo* profile. In contrast to *in vitro* cultured bronchial epithelial cell cultures, *ex vivo* bronchial brush samples consist of a mixed cell population with > 95% of cells from epithelial origin and various different populations of immune cells.^32,33^ While each immune cell population is relatively small, we cannot exclude the potential of these cell populations to influence the overall lipid profile of the *ex vivo* bronchial brush samples. We then compared standard and lipid supplemented *in vitro* culture conditions and observed changes in the PBEC response to RV16 infection identifying clear differences in responses between cultures from non-asthmatic and severe asthmatic donors. Thus, while viral replication was significantly reduced following AA supplementation of *in vitro* differentiated PBECs derived from non-asthmatic donors, we observed a significant increase in viral replication in AA supplemented cultures from severe asthmatic donors. We acknowledge that our study is only able to make comparisons of the response following AA supplementation within each group as we used matched samples. Our study was not powered to make direct comparisons between non-asthmatic subjects and patients with severe asthma, The decrease in viral replication in AA supplemented cultures from non-asthmatic subjects was associated with an indomethacin-sensitive increase in PGE_2_ release and a virus-induced increase in *PTGS2* expression. In contrast, cultures from severe asthmatic donors did not show any changes in the expression of *PTGS2* or PGE_2_ release during RV16 infection and indomethacin did not affect viral replication. Our data suggest that bronchial epithelial cells from asthmatic donors have a deficient PTGS2-PGE_2_ pathway in response to rhinovirus infection which may contribute to virus-induced asthma exacerbations.

Eicosanoids are synthesized by almost all cell types and perform various functions.^19^ PGE_2_ exhibits a wide range of biological activities including regulation of immune responses, tissue repair and homeostasis.^34,35^ Using a mouse model of allergic asthma, it has been shown that airway inflammation is increased in *PTGS2* knockout mice compared to the wildtype, suggesting an anti-inflammatory role of PGE_2_.^36^ In contrast, using a transgenic mouse model, it has been shown that inducible *PTGS2* expression in the respiratory epithelium increased the levels of PGE_2_ in bronchoalveolar lavage fluid and, when the mice were sensitized and challenged using ovalbumin, this resulted in increased IL-4 levels in the lung.^37^ Furthermore, overexpression of PTGS2 in the respiratory epithelium resulted in reduced bacterial clearance.^37^ Similar to studies in animal models, the role of PTGS2/COX2 and PGE_2_ in the airway epithelium of asthmatic subjects is still not fully understood and contradictory data are available.^38^ For example, increased epithelial expression of *PTGS2* was detected in bronchial biopsies from corticosteroid-naive subjects with asthma, while corticosteroid-treated subjects with asthma and non-asthmatic controls showed lower expression levels.^39^ There is also evidence that the expression levels of *PTGS1* and *-2* are altered in cultured bronchial epithelial cells from asthmatic donors,^40^ with cells derived from aspirin-intolerant asthmatic patients showing the lowest expression of *PTGS1* and -*2*. However, beyond these observational studies, mechanistic studies analyzing the role of PTGS2 and PGE_2_ in the human bronchial epithelium in asthma are limited.

Cytosolic phospholipase A2 (cPLA_2_ encoded by *PLA2G4A*), which mobilizes AA from the pool of glycerophospholipids, has been shown to have an increased specificity for AA at the sn-2 position and stearic acid at the sn-1 position,^41^ making PI38:4 (PI18:0/20:4) one of the preferred substrates for cPLA_2_. It has previously been shown that increased baseline release of PGE_2_ in AA supplemented respiratory cell cultures is due to increased availability of endogenous AA rather than increased PTGS activity.^42^ Our studies confirm and extend these findings by showing that AA supplemented bronchial epithelial cultures also had a higher release of PGE_2_ than standard cultures at baseline with no change in *PTGS2* expression. Since we found that AA-supplementation caused an increase in PI38:4 (PI18:0/20:4), it is likely that increased substrate availability for PLA_2_ resulted in enhanced basal release of AA from the lipid membrane pool and conversion into PGE_2_. It has also been shown previously that PGE_2_ is induced in human PBECs during viral infections and is involved in regulating the antiviral host response.^21,43^ In the current study, we found that RV16 infection increased *PTGS2* expression in cultures from non-asthmatic donors and that PTGS-dependent biosynthesis of PGE_2_ was significantly increased in AA supplemented cultures. In contrast, cultures from severe asthmatic subjects did not show increased *PTGS2* expression and PGE_2_ release was unchanged during RV16 infection suggesting that, in asthma, a deficient PGE_2_ pathway in bronchial epithelial cells may contribute to virus-induced asthma exacerbations and delayed recovery. Interestingly, an experimental *in vivo* RV16 infection study in healthy individuals identified increased levels of 15-keto-PGE_2_ that correlated with recovery following RV infection.^44^ Since 15-keto-PGE_2_ is a downstream metabolite of PGE_2_, a deficiency in PGE_2_ in individuals with asthma may result in reduced levels of downstream PGE_2_ metabolites that facilitate recovery and thus prolong symptoms contributing to exacerbations. Using a model that reflects *in vivo* lipid composition of airway epithelial cells has the potential to investigate these mechanisms in more detail.

In the current study, we also observed that RV16-induced release of CXCL8 (IL-8) in AA-supplemented cultures from non-asthmatic donors was dependent on PTGS activity. In contrast, neither *PTGS2* expression nor PGE_2_ release were increased under the same conditions using cells cultured from donors with severe asthma. This suggests that both an increase in *PTGS2* expression and the availability of lipid substrate are required for PGE_2_ to amplify release of IL-8 during viral infection. Consistent with this, it has been shown previously that the presence of AA enhanced the release of IL-6 and CXCL-8 (IL-8) during RV16 infection of a human respiratory epithelial cell line (Beas-2B);^45^ however, changes in the lipid membrane profile as well as viral replication were not analyzed. Our data are also in line with a previous study showing that exogenous PGE_2_ induced the release of CXCL8 (IL-8) in the airway epithelial cell line 16HBE.^46^ Furthermore, it has been shown that binding of PGE_2_ to the EP4 receptor induced a signaling cascade involving PI3K-AKT, CREB and CBP, which results in the formation of a multi-protein complex at the IL-8 promotor region that initiates maximal IL-8 expression.^47^ In contrast, using macrophages, it has been shown that PGE_2_ suppressed LPS-induced inflammatory responses by modulating the expression of inflammatory genes via targeting inflammatory gene enhancers.^25^ These data suggest that the role of lipid mediators is cell type and environment specific, highlighting the importance of considering the lipid membrane composition and the availability of AA for eicosanoid synthesis in cellular models for mechanistic studies. Increasing the availability of AA in the lipid membrane can enhance lipid-dependent signaling events. It is important to note that PUFAs are involved in many other signaling pathways, e.g. PI3 kinases, as well as cellular processes involving protein trafficking, localization, conformation and activation.

In summary, our results suggest that standard cultures of *in vitro* differentiated PBECs are deficient in PUFAs and show reduced biosynthesis of eicosanoids due to limited availability of lipid precursors. Critically, this alters the overall antiviral immune response of the cultured epithelial cells; furthermore, we identified disease-related differences in these responses. We therefore recommend that the lipid membrane profile of *in vitro* cultured differentiated PBECs is adjusted to more closely resemble the *in vivo* profile by supplementing the culture medium with PUFAs, especially arachidonic acid. This will help ensure that *in vitro* culture models have the potential to respond with an orchestrated signaling cascade to exogenous stimuli, e.g. during virus infections, that includes the biosynthesis of lipid mediators. Furthermore, this will also allow the identification of disease-related differences, e.g. altered lipid-dependent signaling during viral infections in asthma. Improving human *in vitro* culture models to reflect the *in vivo* situation as closely as possible, will facilitate the translation of results into the *in vivo* situation, an essential aspect in research and drug development.

## Supplementary Material

Panchal et al supplementary tables.docx

Panchal et al supplementary figures.pdf
