# Supplementary material for: Membrane lipid composition of bronchial epithelial cells influences antiviral responses during rhinovirus infection
Source: Tissue Barriers. 2024 Jan 5;12(4):2300580. doi: 10.1080/21688370.2023.2300580 (PMC11583602; doi:10.1080/21688370.2023.2300580)
Supplement: Panchal et al supplementary tables.docx [file KTIB_A_2300580_SM9032.docx]

**Supplementary Table 1: Clinical characteristics of volunteers without and with severe asthma included into this study that provided bronchial brush samples for the culture of bronchial epithelial cells and/or lipidomic analysis.**

| Parameter | Control group without asthma | Subjects with severe asthma | P value |
| --- | --- | --- | --- |
| Gender (F/M) | 10/12 | 11/3 | - |
| Age | 30 (23-50) | 43.5 (35.25-57.25) | 0.0695 |
| BMI | 26.47 (23.59-31.97) | 31.91 (36.49-39.85) | 0.0414* |
| Skin prick test positive/negative/no data | 15/5/2 | 12/2 | - |
| Pre FEV1% predicted | 107.4 (103.0-117.7) | 91.75 (67.40-104.7) | 0.0054** |

Data shown are medians (inter-quartile range). Data were compared using the Mann-Whitney U test. *p<0.05, **p<0.01.

**Supplementary Table 2:** Internal standard composition of 50µl added to each sample during extraction procedure.

| **Standard Name** | **Amount (nmol)** |
| --- | --- |
| Dimyristoyl phosphatidylcholine (DMPC) | 5.0 |
| Dimyristoyl phosphatidylethanolamine (DMPE) | 2.0 |
| Dimyristoyl phosphatidylserine (DMPS) | 1.0 |
| Dimyristoyl phosphatidylglycerol (DMPG) | 1.0 |
| Dimyristoyl phosphatidic acid **(**DMPA) | 0.5 |
| Lysophosphatidylcholine (LPC17:0) | 0.5 |
| Sphingomyelin (SM16:0) | 0.5 |
| Ceramide 12:0 | 1.0 |
| Triacylglycerol (TAG) | 5.0 |

**Supplementary Table 3:** Molecular lipid species and their corresponding mass used to identify phospholipids in samples.

| Generic formula | Structure^*^ | Mass |
| --- | --- | --- |
| PHOSPHATIDYLCHOLINE | | |
| PC30:0 | PC16:0/14:0 | 706 |
| PC32:0 | PC16:0/16:0 | 734 |
| PC32:1 | PC16:0/16:1 | 732 |
| PC34:1 | PC16:0/18:1 | 760 |
| PC34:2 | PC16:0/18:2 | 758 |
| PC36:1 | PC18:0/18:1 | 788 |
| PC36:2 | PC18:0/18:2 | 786 |
| PC36:3 | PC18:1/18:2 | 784 |
| PC36:4 | PC16:0/20:4 | 782 |
| PC38:2 | PC18:0/20:2 | 814 |
| PC38:3 | PC18:0/20:3 | 812 |
| PC38:4 | PC18:0/20:4 | 810 |
| PC38:5 | PC18:1/20:4 | 808 |
| PC38:6 | PC16:0/22:6 | 806 |
| PC40:6 | PC18:0/22:6 | 834 |
| Others | | |
| PC32a:0 | PC16:0a/16:0 | 720 |
| PC32a:1 | PC18:0a/14:1 | 718 |
| PC34a:1 | PC16:0a/18:1 | 746 |
| PC34a:2 | PC16:0a/18:2 | 744 |
| Generic formula | **Structure*** | **Mass** |
| PHOSPHOINOSITOL | | |
| PI32:0 | PI16:0/16:0 | 809 |
| PI32:1 | PI16:1/16:0 | 807 |
| PI34:1 | PI16:0/18:1 | 835 |
| PI34:2 | PI16:0/18:2 | 833 |
| PI36:1 | PI18:0/18:1 | 863 |
| PI36:2 | PI18:0/18:2 | 861 |
| PI36:3 | PI18:1/18:2 | 859 |
| PI36:4 | PI16:0/20:4 | 857 |
| PI38:3 | PI18:0/20:3 | 887 |
| PI38:4 | PI18:0/20:4 | 885 |

| Generic formula | Structure^*^ | Mass |
| --- | --- | --- |
| PHOSPHATIDYLETHANOLAMINE | | |
| PE32:1 | PE16:0/16:1 | 690 |
| PE34:1 | PE16:0/18:1 | 718 |
| PE34:2 | PE16:0/18:2 | 716 |
| PE36:1 | PE18:0/18:1 | 746 |
| PE36:2 | PE18:1/18:1 | 744 |
| PE36:3 | PE18:1/18:2 | 742 |
| PE38:2 | PE18:0/20:2 | 772 |
| PE38:3 | PE18:0/20:3 | 770 |
| PE38:4 | PE18:0/20:4 | 768 |
| PE38:5 | PE18:1/20:4 | 766 |
| PE38:6 | PE16:0/22:6 | 764 |
| PE40:3 | PE18:0/22:3 | 798 |
| PE40:4 | PE18:0/22:4 | 796 |
| PE40:5 | PE18:0/22:5 | 794 |
| PE40:6 | PE18:0/22:6 | 792 |
| PE40:7 | PE18:1/22:6 | 790 |
| Generic formula | **Structure*** | **Mass** |
| PHOSPHATIDYLSERINE | | |
| PS36:1 | PS18:0/18:1 | 788 |
| PS38:3 | PS18:0/20:3 | 812 |
| PS40:4 | PS18:0/22:4 | 838 |
| PS40:5 | PS18:0/22:5 | 836 |
| PS40a:8 | PS20:4a/20:4 | 816 |
| PS40a:9 | PS20:5a//20:4 | 814 |
| PS42a:8 | PS20:3a/22:5 | 844 |
| PS42a:9 | PS20:4a/22:5 | 842 |
| PS42a:10 | PS22:6a/20:4 | 840 |
| Generic formula | **Structure*** | **Mass** |
| PHOSPHATIDYLGLYCEROL | | |
| PG32:0 | PG16:0/16:0 | 721 |
| PG34:1 | PG16:0/18:1 | 747 |
| PG36:2 | PG18:1/18:1 | 773 |
| PG36:3 | PG18:1/18:2 | 771 |

*Mass spectrometry provided a generic formula based on total number of carbon atoms and unsaturated double bonds in each lipid class. Based on previous analyses and MS/MS fragmentation in negative ionisation to generate diagnostic fatty acyl residues, column 2 identifies the most probable molecular structures. Here designations are given as PXa:y/b:x for each phospholipid class, where X represents the headgroup base (e.g. choline or glycerol), A and B are number of carbon atoms in the sn-1 and sn-2 fatty acyl residues and y and x are numbers of double bonds.

**Supplementary Table 4:** Details of primers used for qPCR.

| **Gene name** | **Product code** | **Fluorescent probe** | **Manufacturer** |
| --- | --- | --- | --- |
| UBC/GAPDH duplex |  | Cy5 and FAM | PrimerDesign, Southampton, UK |
| PTGS1 | Hs00377726_m1 | FAM-MGB | ThermoFisher Scientific |
| PTGS2 | Hs00153133_m1 | FAM-MGB | ThermoFisher Scientific |
| PTGES | Hs00610420_m1 | FAM-MGB | ThermoFisher Scientific |
| PLA2G4A | Hs00996912_m1 | FAM-MGB | ThermoFisher Scientific |

**Supplementary Table 5:** Lipid membrane profile of *ex vivo* human bronchial brushings (*ex vivo*) and matching *in vitro* differentiated PBECs of 5 independent subjects without asthma. Statistical analysis was performed using 2-way RM ANOVA with Bonferroni’s multiple comparisons test.

|  | *ex vivo* | | | | *in vitro* PBECs | |  |
| --- | --- | --- | --- | --- | --- | --- | --- |
| Phosphatidylcholine | | | | | | | |
|  | **MEAN** | **SEM** | | | **MEAN** | **SEM** | **P value** |
| PC30:0 | **0.69** | | **0.097** | | **2.89** | **0.203** | **0.0125** |
| PC32:0 | 7.43 | | 1.102 | | 4.97 | 0.511 | >0.9999 |
| PC32:1 | **1.24** | | **0.105** | | **9.28** | **0.504** | **0.0010** |
| PC34:1 | 24.81 | | 1.326 | | 33.89 | 1.376 | 0.2690 |
| PC34:2 | 12.26 | | 0.908 | | 9.11 | 0.416 | 0.8515 |
| PC36:1 | 9.19 | | 0.734 | | 5.34 | 0.342 | 0.1824 |
| PC36:2 | 14.29 | | 0.354 | | 19.80 | 1.377 | 0.3644 |
| PC36:3 | **6.57** | | **0.323** | | **3.33** | **0.136** | **0.0038** |
| PC36:4 | **6.22** | | **0.344** | | **0.60** | **0.174** | **0.0037** |
| PC38:2 | 0.89 | | 0.092 | | 1.94 | 0.198 | 0.2000 |
| PC38:3 | 3.18 | | 0.175 | | 2.43 | 0.084 | 0.5027 |
| PC38:4 | **7.39** | | **0.339** | | **1.15** | **0.180** | **0.0014** |
| PC38:6 | **1.75** | | **0.135** | | **0.06** | **0.006** | **0.0048** |
| PC40:6 | **1.29** | | **0.089** | | **0.09** | **0.019** | **0.0042** |
| Others |  | |  | |  |  |  |
| PC32a:0 | 0.71 | | 0.067 | | 0.80 | 0.125 | 0.0790 |
| PC32a:1 | 0.21 | | 0.011 | | 0.92 | 0.113 | >0.9999 |
| PC34a:1 | 1.21 | | 0.062 | | 2.09 | 0.217 | 0.3524 |
| PC34a:2 | 0.67 | | 0.060 | | 1.29 | 0.149 | 0.5857 |
|  | ***ex vivo*** | | | | ***in vitro* PBECs** | |  |
| Phosphoinositol | | | | | | | |
|  | **MEAN** | | **SEM** | | **MEAN** | **SEM** | **P value** |
| PI32:0 | 1.43 | | | 0.085 | 0.73 | 0.071 | 0.0645 |
| PI32:1 | **0.49** | | | **0.101** | **1.65** | **0.196** | **0.0247** |
| PI34:1 | 10.99 | | | 0.579 | 12.58 | 1.402 | >0.9999 |
| PI34:2 | 4.51 | | | 0.668 | 4.48 | 0.322 | >0.9999 |
| PI36:1 | 11.01 | | | 0.765 | 13.42 | 2.194 | >0.9999 |
| PI36:2 | 11.03 | | | 0.618 | 16.60 | 1.558 | 0.1672 |
| PI36:3 | 3.41 | | | 0.220 | 5.11 | 0.766 | 0.5432 |
| PI36:4 | **7.01** | | | **0.157** | **1.25** | **0.427** | **0.0023** |
| PI38:3 | **10.13** | | | **0.445** | **33.00** | **2.303** | **0.0057** |
| PI38:4 | **39.99** | | | **1.084** | **11.17** | **2.327** | **0.0025** |
|  | | | | | | | |

|  | *ex vivo* | | | *in vitro* PBECs | |  |
| --- | --- | --- | --- | --- | --- | --- |
| phosphatidylethanolamine | | | | | | |
|  | **MEAN** | | **SEM** | **MEAN** | **SEM** | **P value** |
| PE32:1 | **0.10** | | **0.043** | **1.56** | **0.067** | **0.0004** |
| PE34:1 | **6.35** | | **0.356** | **14.21** | **0.478** | **0.0014** |
| PE34:2 | **2.43** | | **0.315** | **5.30** | **0.423** | **0.0324** |
| PE36:1 | 22.50 | | 1.085 | 14.69 | **2.306** | 0.4289 |
| PE36:2 | **13.45** | | **0.299** | **31.36** | **2.124** | **0.0215** |
| PE36:3 | 3.78 | | 0.318 | 4.63 | 0.318 | >0.9999 |
| PE38:2 | 4.48 | | 0.326 | 3.69 | 1.024 | >0.9999 |
| PE38:3 | 11.60 | | 0.265 | 7.46 | 1.472 | 0.7060 |
| PE38:4 | 4.32 | | 0.941 | 4.71 | 2.447 | >0.9999 |
| PE38:5 | **1.53** | | **0.137** | **0.25** | **0.254** | **0.0405** |
| PE38:6 | 3.70 | | 0.471 | 1.02 | 0.713 | 0.4808 |
| PE40:3 | 2.63 | | 0.216 | 1.52 | 0.287 | 0.2395 |
| PE40:4 | 1.55 | | 0.178 | 1.34 | 0.619 | >0.9999 |
| PE40:5 | 1.60 | | 0.171 | 1.46 | 1.048 | >0.9999 |
| PE40:6 | 3.48 | | 0.425 | 3.89 | 2.743 | >0.9999 |
| PE40:7 | **12.11** | | **0.579** | **2.60** | **1.045** | **0.0010** |
|  | ***ex vivo*** | | | ***in vitro* PBECs** | |  |
| phosphatidylserine | | | | | | |
|  | **MEAN** | **SEM** | | **MEAN** | **SEM** | **P value** |
| PS36:1 | 72.76 | | 1.058 | 66.18 | 1.478 | 0.2371 |
| PS38:3 | **7.91** | | **0.689** | **4.26** | **0.575** | **0.0126** |
| PS40:4 | **3.46** | | **0.218** | **1.63** | **0.302** | **0.0140** |
| PS40:5 | **5.77** | | **0.395** | **1.05** | **0.203** | **0.0107** |
| PS40a:8 | 2.05 | | 0.178 | 2.54 | 0.386 | >0.9999 |
| PS40a:9 | **4.25** | | **0.211** | **13.24** | **0.513** | **0.0002** |
| PS42a:8 | 0.78 | | 0.120 | 2.05 | 0.216 | 0.0659 |
| PS42a:9 | **2.45** | | **0.099** | **4.66** | **0.240** | **0.0120** |
| PS42a:10 | **0.56** | | **0.112** | **4.38** | **0.555** | **0.0401** |
|  | ***ex vivo*** | | | ***in vitro* PBECs** | |  |
| Phosphatidylglycerol | | | | | | |
|  | **MEAN** | | **SEM** | **MEAN** | **SEM** | **P value** |
| PG32:0 | 6.18 | | 1.062 | 3.92 | 1.735 | 0.4770 |
| PG34:1 | 23.32 | | 2.082 | 23.85 | 3.047 | >0.9999 |
| PG36:2 | 15.32 | | 2.965 | 25.15 | 5.023 | 0.4211 |
| PG36:3 | **10.10** | | **1.315** | **14.25** | **1.903** | **0.0486** |

**Supplementary Table 6:** **PUFA supplementation of culture medium alters the lipid membrane profile of *in vitro* differentiated human primary bronchial epithelial cells from non-asthmatic subjects.** Matching *in vitro* differentiated PBECs of 3-5 independent non-asthmatic subjects were supplemented with arachidonic acid (AA), linoleic acid (LA) or docosahexaenoic acid (DHA) as described in the methods section and the lipid profile analysed by mass spectrometry. Significance was tested against the un-supplemented *in vitro* culture using 2-way RM ANOVA with Bonferroni’s multiple comparisons test. b.d.: below detection limit

|  | *in vitro* (n=5) | *in vitro* +AA (n=5) | | *in vitro* +LA (n=4) | | *in vitro* +DHA (n=3) | | *ex vivo* (n=5) |
| --- | --- | --- | --- | --- | --- | --- | --- | --- |
| Phosphatidylcholine | | | | | | | | |
|  | **MEAN±SEM** | **MEAN±SEM** | **P value** | **MEAN±SEM** | **P value** | **MEAN±SEM** | **P value** | **MEAN±SEM** |
| PC30:0 | **2.89±0.203** | **3.84±0.235** | **0.033** | 3.40±0.222 | 0.820 | 3.16±0.488 | >0.999 | 0.69±0.097 |
| PC32:0 | **4.97±0.511** | **7.94±0.429** | **0.020** | **7.20±0.556** | **0.045** | 5.97±0.597 | 0.498 | 7.43±1.102 |
| PC32:1 | 9.28±0.504 | 9.55±0.412 | >0.999 | 8.01±0.254 | 0.578 | 8.57±0.653 | 0.385 | 1.24±0.105 |
| PC34:1 | 33.89±1.376 | 34.31±1.350 | >0.999 | 30.93±1.694 | 0.776 | 37.44±1.905 | 0.665 | 24.81±1.326 |
| PC34:2 | 9.11±0.416 | 7.55±0.382 | 0.155 | 10.55±0.694 | 0.850 | 7.35±0.360 | 0.466 | 12.26±0.908 |
| PC36:1 | 5.34±0.342 | 4.95±0.293 | >0.999 | 4.33±0.273 | 0.070 | 5.86±0.554 | >0.999 | 9.19±0.734 |
| PC36:2 | 19.80±1.377 | 15.74±0.749 | 0.063 | 15.43±0.360 | 0.092 | 17.42±1.084 | 0.596 | 14.29±0.354 |
| PC36:3 | 3.33±0.136 | 3.12±0.396 | >0.999 | **6.82±0.673** | **0.027** | 2.45±0.415 | 0.338 | 6.57±0.323 |
| PC36:4 | 0.60±0.174 | 2.22±0.431 | 0.070 | 1.49±0.254 | 0.085 | 0.64±0.141 | >0.999 | 6.22±0.344 |
| PC38:2 | 1.94±0.198 | 1.36±0.074 | 0.114 | 1.75±0.112 | >0.999 | 2.57±0.311 | >0.999 | 0.89±0.092 |
| PC38:3 | 2.43±0.084 | 2.19±0.162 | 0.672 | 2.59±0.227 | >0.999 | 1.88±0.219 | 0.545 | 3.18±0.175 |
| PC38:4 | **1.15±0.180** | **2.14±0.256** | **0.034** | **2.33±0.283** | **0.044** | 0.83±0.142 | 0.906 | 7.39±0.339 |
| PC38:6 | 0.06±0.006 | 0.12±0.018 | 0.087 | 0.13±0.032 | 0.409 | 1.28±0.362 | 0.253 | 1.75±0.135 |
| PC40:6 | **0.09±0.019** | **0.23±0.034** | **0.011** | 0.14±0.032 | 0.494 | 0.40±0.074 | 0.159 | 1.29±0.089 |
| Others |  |  |  |  |  |  |  |  |
| PC32a:0 | 0.80±0.125 | 0.96±0.114 | 0.957 | 1.05±0.182 | 0.180 | 0.60±0.103 | 0.885 | 0.71±0.067 |
| PC32a:1 | 0.92±0.113 | 0.86±0.090 | 0.669 | 0.81±0.132 | 0.024 | 0.76±0.237 | 0.780 | 0.21±0.011 |
| PC34a:2 | 1.29±0.149 | 1.06±0.150 | 0.496 | 1.32±0.179 | 0.229 | 1.31±0.255 | 0.295 | 0.67±0.060 |
| PC34a:1 | 2.09±0.217 | 1.85±0.165 | 0.320 | 1.74±0.204 | >0.999 | 1.50±0.198 | >0.999 | 1.21±0.062 |
|  | ***in vitro* (n=5)** | ***in vitro* +AA (n=5)** | | ***in vitro* +LA (n=4)** | | ***in vitro* +DHA (n=3)** | | ***ex vivo* (n=5)** |
| Phosphoinositol | | | | | | | | |
|  | **MEAN±SEM** | **MEAN±SEM** | **P value** | **MEAN±SEM** | **P value** | **MEAN±SEM** | **P value** | **MEAN±SEM** |
| PI32:0 | 0.73±0.070 | 0.89±0.101 | >0.999 | 0.92±0.182 | >0.999 | 1.57±0.292 | 0.538 | 1.43±0.085 |
| PI32:1 | 1.66±0.195 | 1.63±0.137 | >0.999 | 1.45±0.420 | >0.999 | 2.03±0.225 | >0.999 | 0.49±0.101 |
| PI34:1 | 12.58±1.402 | 12.70±1.158 | >0.999 | 11.13±1.898 | >0.999 | 14.86±0.379 | >0.999 | 10.99±0.579 |
| PI34:2 | 4.48±0.321 | 3.00±0.365 | 0.151 | 3.76±0.340 | >0.999 | 3.80±0.554 | >0.999 | 4.51±0.668 |
| PI36:1 | 13.42±2.194 | 11.14±1.404 | 0.262 | 11.24±1.470 | >0.999 | 14.99±2.011 | >0.999 | 11.02±0.766 |
| PI36:2 | 16.61±1.558 | 11.55±0.705 | 0.111 | 12.96±0.987 | 0.730 | 15.56±0.183 | >0.999 | 11.03±0.618 |
| PI36:3 | 5.11±0.766 | 4.86±0.283 | >0.999 | 6.57±0.711 | 0.681 | 6.52±0.805 | 0.594 | 3.41±0.221 |
| PI36:4 | 1.25±0.427 | 3.23±0.575 | 0.182 | 1.45±0.369 | >0.999 | 1.34±0.097 | >0.999 | 7.01±0.157 |
| PI38:3 | 33.00±2.304 | 24.77±1.643 | 0.192 | 34.70±2.746 | >0.999 | 31.33±1.188 | >0.999 | 10.13±0.446 |
| PI38:4 | **11.17±2.327** | **26.22±2.993** | **0.023** | 15.82±2.700 | >0.999 | 7.98±0.459 | >0.999 | 39.99±1.084 |

**Supplementary Table 6 (continued):** **PUFA supplementation of culture medium alters the lipid membrane profile of *in vitro* differentiated human primary bronchial epithelial cells from non-asthmatic subjects.** Matching *in vitro* differentiated PBECs of 3-5 independent non-asthmatic subjects were supplemented with arachidonic acid (AA), linoleic acid (LA) or docosahexaenoic acid (DHA) as described in the methods section and the lipid profile analysed by mass spectrometry. Significance was tested against the un-supplemented in vitro culture using 2-way RM ANOVA with Bonferroni’s multiple comparisons test. b.d.: below detection limit

|  | *in vitro* (n=5) | | | | *in vitro* +AA (n=5) | | | | | *in vitro* +LA (n=4) | | | | *in vitro* +DHA (n=3) | | | | *ex vivo* (n=5) | |  |  |
| --- | --- | --- | --- | --- | --- | --- | --- | --- | --- | --- | --- | --- | --- | --- | --- | --- | --- | --- | --- | --- | --- |
| phosphatidylethanolamine | | | | | | | | | | | | | | | | | | | | |  |
|  | **MEAN±SEM** | | **MEAN±SEM** | | | | **P value** | | **MEAN±SEM** | | **P value** | | **MEAN±SEM** | | **P value** | | **MEAN±SEM** | |  |  |  |
| PE32:1 | 1.56±0.067 | | 1.63±0.058 | | | | >0.999 | | 1.44±0.158 | | >0.999 | | 1.38±0.224 | | 0.978 | | 0.10±0.041 | |  |  |  |
| PE34:1 | 14.21±0.478 | | 13.10±0.294 | | | | 0.100 | | 12.88±0.572 | | 0.204 | | 13.45±0.693 | | >0.999 | | 6.35±0.356 | |  |  |  |
| PE34:2 | 5.30±0.423 | | 3.93±0.181 | | | | 0.149 | | 4.85±0.321 | | 0.924 | | 3.29±0.376 | | 0.214 | | 2.43±0.311 | |  |  |  |
| PE36:1 | 14.69±2.306 | | 18.33±0.771 | | | | 0.630 | | 17.33±0.814 | | >0.999 | | 16.67±2.293 | | >0.999 | | 22.50±1.085 | |  |  |  |
| PE36:2 | 31.36±2.124 | | 23.92±1.184 | | | | 0.115 | | 26.14±0.592 | | 0.212 | | 20.63±2.029 | | 0.199 | | 13.45±0.300 | |  |  |  |
| PE36:3 | 4.63±0.318 | | 3.91±0.313 | | | | 0.947 | | **7.08±0.474** | | **0.024** | | 3.13±0.340 | | 0.463 | | 3.78±0.319 | |  |  |  |
| PE38:2 | 3.69±1.024 | | 6.07±0.697 | | | | 0.393 | | 4.84±1.137 | | >0.999 | | 3.83±0.134 | | >0.999 | | 4.48±0.325 | |  |  |  |
| PE38:3 | 7.46±1.472 | | 8.22±0.799 | | | | >0.999 | | 8.94±1.085 | | >0.999 | | 12.10±2.031 | | >0.999 | | 11.60±0.265 | |  |  |  |
| PE38:4 | 4.71±2.447 | | 7.24±1.377 | | | | 0.634 | | 3.42±1.109 | | >0.999 | | 0.10±0.103 | | >0.999 | | 4.33±0.941 | |  |  |  |
| PE38:5 | 0.25±0.254 | | 1.71±0.509 | | | | 0.216 | | 0.27±0.226 | | >0.999 | | b.d. | | --- | | 1.53±0.137 | |  |  |  |
| PE38:6 | 1.02±0.713 | | 0.43±0.168 | | | | >0.999 | | 0.83±0.606 | | >0.999 | | 3.85±0.559 | | 0.246 | | 3.70±0.471 | |  |  |  |
| PE40:3 | 1.52±0.287 | | 1.65±0.183 | | | | >0.999 | | 1.66±0.274 | | >0.999 | | 2.56±0.402 | | 0.804 | | 2.63±0.214 | |  |  |  |
| PE40:4 | 1.34±0.619 | | 2.32±0.336 | | | | 0.697 | | 0.66±0.383 | | >0.999 | | 4.23±1.173 | | 0.826 | | 1.55±0.177 | |  |  |  |
| PE40:5 | 1.46±1.048 | | 0.82±0.302 | | | | >0.999 | | 0.60±0.509 | | >0.999 | | b.d. | | --- | | 1.60±0.171 | |  |  |  |
| PE40:6 | 3.89±2.743 | | 1.20±0.218 | | | | >0.999 | | 1.70±0.466 | | >0.999 | | 1.13±0.631 | | >0.999 | | 3.48±0.425 | |  |  |  |
| PE40:7 | 2.60±1.045 | | 4.58±0.960 | | | | 0.160 | | 6.53±1.275 | | 0.465 | | 7.68±0.587 | | 0.332 | | 12.11±0.578 | |  |  |  |
|  | ***in vitro*** | | ***in vitro*+AA** | | | | | | ***in vitro*+LA** | | | | | ***in vitro*+DHA** | | | | ***ex vivo*** | | | |
| phosphatidylserine | | | | | | | | | | | | | | | | | | | | |  |
|  | **MEAN±SEM** | | | **MEAN±SEM** | | | **P value** | | **MEAN±SEM** | | **P value** | | | **MEAN±SEM** | | **P value** | | **MEAN±SEM** | |  |  |
| PS36:1 | 66.18±1.478 | | | 67.78±1.548 | | | 0.905 | | 63.52±3.821 | | >0.999 | | | 71.51±1.907 | | 0.102 | | 72.77±1.058 | |  |  |
| PS38:3 | 4.26±0.575 | | | 5.60±0.826 | | | 0.091 | | 9.64±2.019 | | 0.111 | | | 3.68±0.885 | | 0.427 | | 7.91±0.687 | |  |  |
| PS40:4 | **1.63±0.302** | | | **3.07±0.557** | | | **0.043** | | 2.18±0.515 | | 0.208 | | | 0.64±0.289 | | 0.138 | | 3.46±0.218 | |  |  |
| PS40:5 | 1.05±0.203 | | | 1.09±0.173 | | | >0.999 | | 0.90±0.118 | | >0.999 | | | 0.78±0.293 | | 0.940 | | 5.77±0.395 | |  |  |
| PS40a:8 | 2.54±0.386 | | | 2.73±0.261 | | | >0.999 | | 2.62±0.261 | | >0.999 | | | 3.00±0.314 | | >0.999 | | 2.05±0.178 | |  |  |
| PS40a:9 | **13.24±0.513** | | | **9.78±0.611** | | | **0.031** | | **9.45±0.551** | | **0.021** | | | 9.99±0.116 | | 0.191 | | 4.25±0.211 | |  |  |
| PS42a:8 | 2.05±0.216 | | | 1.80±0.259 | | | >0.999 | | 3.19±0.914 | | >0.999 | | | 2.60±0.447 | | >0.999 | | 0.78±0.120 | |  |  |
| PS42a:9 | 4.66±0.240 | | | 3.84±0.238 | | | 0.170 | | 4.48±0.444 | | >0.999 | | | 4.57±0.196 | | >0.999 | | 2.45±0.100 | |  |  |
| PS42a:10 | 4.38±0.555 | | | 4.31±0.575 | | | >0.999 | | 4.03±0.736 | | >0.999 | | | 3.22±0.341 | | 0.845 | | 0.56±0.112 | |  |  |
|  | ***in vitro*** | | | ***in vitro*+AA** | | | | | ***in vitro*+LA** | | | | | ***in vitro*+DHA** | | | | ***ex vivo*** | |  |  |
| Phosphatidylglycerol | | | | | | | | | | | | | | | | | | | |  |  |
|  | | **MEAN±SEM** | | | | **MEAN±SEM** | | **P value** | | **MEAN±SEM** | | **P value** | | **MEAN±SEM** | | **P value** | | **MEAN**±SEM | |  |  |
| PG32:0 | | 3.92±1.736 | | | | 7.61±0.967 | | 0.675 | | **7.24±2.026** | | **0.025** | | 9.09±0.924 | | >0.999 | | 6.18±1.062 | |  |  |
| PG34:1 | | 23.85±3.046 | | | | 32.24±3.165 | | 0.570 | | 24.57±1.381 | | >0.999 | | 30.06±4.373 | | >0.999 | | 23.32±2.082 | |  |  |
| PG36:2 | | 25.15±5.022 | | | | 21.27±2.511 | | >0.999 | | 16.71±1.610 | | >0.999 | | 21.44±4.729 | | >0.999 | | 15.31±2.964 | |  |  |
| PG36:3 | | 14.25±1.903 | | | | 15.37±3.470 | | >0.999 | | 11.88±2.288 | | >0.999 | | 4.97±2.578 | | 0.109 | | 10.10±1.313 | |  |  |

**Supplementary Table 7:** Lipid membrane profile of *ex vivo* bronchial brushings (*ex vivo*) and matching *in vitro* differentiated PBECs of subjects with severe asthma. *Ex vivo* n=6; *in vitro* n=4. Statistical analysis was performed using 2-way RM ANOVA with Bonferroni’s multiple comparisons test. b.d.: below detection limit

|  | *ex vivo* | | | | *in vitro* PBECs | |  |
| --- | --- | --- | --- | --- | --- | --- | --- |
| Phosphatidylcholine | | | | | | | |
|  | **MEAN** | **SEM** | | | **MEAN** | **SEM** | **P value** |
| PC30:0 | 1.66 | | 0.511 | | 3.17 | 0.295 | >0.9999 |
| PC32:0 | 12.45 | | 2.86 | | 5.605 | 0.671 | >0.9999 |
| PC32:1 | 2.3 | | 0.661 | | 9.24 | 0.675 | 0.2335 |
| PC34:1 | 23.25 | | 0.848 | | 31.11 | 0.775 | 0.3022 |
| PC34:2 | 11.82 | | 0.701 | | 9.888 | 0.177 | 0.9141 |
| PC36:1 | 7.66 | | 0.66 | | 5.103 | 0.551 | >0.9999 |
| PC36:2 | 12.06 | | 1.141 | | 20.8 | 0.888 | 0.2186 |
| PC36:3 | 6.76 | | 0.578 | | 3.373 | 0.089 | 0.1600 |
| PC36:4 | 5.65 | | 0.55 | | 0.4575 | 0.085 | 0.0841 |
| PC38:2 | 0.88 | | 0.148 | | 1.87 | 0.209 | 0.6632 |
| PC38:3 | 3.32 | | 0.472 | | 2.533 | 0.21 | >0.9999 |
| PC38:4 | 6.53 | | 0.695 | | 0.95 | 0.011 | 0.1791 |
| PC38:6 | **1.66** | | **0.082** | | **0.0675** | **0.013** | **0.0042** |
| PC40:6 | **1.1** | | **0.103** | | **0.09** | **0.019** | **0.0215** |
| Others |  | |  | |  |  |  |
| PC32a:0 | 0.26 | | 0.029 | | 1.105 | 0.141 | 0.1525 |
| PC32a:1 | 0.86 | | 0.108 | | 0.91 | 0.159 | >0.9999 |
| PC34a:1 | 1.12 | | 0.05 | | 2.225 | 0.254 | 0.3064 |
| PC34a:2 | 0.68 | | 0.082 | | 1.51 | 0.151 | 0.0673 |
|  | ***ex vivo*** | | | | ***in vitro* PBECs** | |  |
| Phosphoinositol | | | | | | | |
|  | **MEAN** | | **SEM** | | **MEAN** | **SEM** | **P value** |
| PI32:0 | 1.38 | | | 0.298 | 0.68 | 0.081 | >0.9999 |
| PI32:1 | **0.49** | | | **0.03** | **1.69** | **0.079** | **0.0016** |
| PI34:1 | 11.19 | | | 0.717 | 11.02 | 1.167 | >0.9999 |
| PI34:2 | 4.08 | | | 0.532 | 4.08 | 0.297 | >0.9999 |
| PI36:1 | 11.25 | | | 0.814 | 12.18 | 2.027 | >0.9999 |
| PI36:2 | 11.14 | | | 0.826 | 18.07 | 1.604 | 0.0881 |
| PI36:3 | 4.07 | | | 0.691 | 5.49 | 0.378 | >0.9999 |
| PI36:4 | **6.19** | | | **0.523** | **0.91** | **0.17** | **0.0317** |
| PI38:3 | **12.94** | | | **2.186** | **35.03** | **3.268** | **0.0490** |
| PI38:4 | **37.27** | | | **3.273** | **10.86** | **2.194** | **0.0438** |

|  | *ex vivo* | | | *in vitro* PBECs | |  |
| --- | --- | --- | --- | --- | --- | --- |
| phosphatidylethanolamine | | | | | | |
|  | **MEAN** | | **SEM** | **MEAN** | **SEM** | **P value** |
| PE32:1 | **0.21** | | **0.051** | **1.88** | **0.083** | **0.0172** |
| PE34:1 | 7.7 | | 0.493 | 14.77 | 0.409 | 0.0553 |
| PE34:2 | **2.83** | | **0.187** | **5.75** | **0.118** | **0.0241** |
| PE36:1 | 21.78 | | 0.865 | 15.9 | 2.375 | 0.2596 |
| PE36:2 | **12.09** | | **0.716** | **33.03** | **0.974** | **0.0035** |
| PE36:3 | 4.47 | | 0.365 | 4.9 | 0.269 | >0.9999 |
| PE38:2 | 4.59 | | 0.23 | 3.8 | 1.275 | >0.9999 |
| PE38:3 | 12.32 | | 0.786 | 6.56 | 0.703 | 0.2829 |
| PE38:4 | 3.22 | | 0.902 | 2.12 | 1.243 | >0.9999 |
| PE38:5 | 1.92 | | 0.345 | b.d. | --- | --- |
| PE38:6 | 3.62 | | 0.481 | 0.82 | 0.151 | 0.3413 |
| PE40:3 | 2.8 | | 0.4 | 0.92 | 0.362 | 0.5988 |
| PE40:4 | 1.65 | | 0.303 | 1.05 | 0.291 | >0.9999 |
| PE40:5 | 1.39 | | 0.347 | 1.89 | 1.776 | >0.9999 |
| PE40:6 | 2.07 | | 0.408 | 3.42 | 1.966 | >0.9999 |
| PE40:7 | **12.35** | | **0.859** | **2.76** | **0.95** | **0.0500** |
|  | ***ex vivo*** | | | ***in vitro* PBECs** | |  |
| phosphatidylserine | | | | | | |
|  | **MEAN** | **SEM** | | **MEAN** | **SEM** | **P value** |
| PS36:1 | 65.71 | | 2.42 | 65.58 | 2.77 | >0.9999 |
| PS38:3 | 11.29 | | 1.679 | 5.69 | 0.614 | 0.9165 |
| PS40:4 | 4.51 | | 0.792 | 1.65 | 0.298 | 0.0653 |
| PS40:5 | 7.13 | | 1.114 | 0.87 | 0.2 | 0.1275 |
| PS40a:8 | 2.3 | | 0.113 | 2.41 | 0.297 | >0.9999 |
| PS40a:9 | 4.52 | | 0.386 | 12.59 | 1.78 | 0.1416 |
| PS42a:8 | 1.09 | | 0.144 | 1.58 | 0.291 | >0.9999 |
| PS42a:9 | 2.75 | | 0.17 | 4.95 | 0.438 | 0.1249 |
| PS42a:10 | 0.7 | | 0.139 | 4.69 | 0.698 | 0.0615 |
|  | ***ex vivo* PBECs** | | | ***in vitro* PBECs** | |  |
| Phosphatidylglycerol | | | | | | |
|  | **MEAN** | | **SEM** | **MEAN** | **SEM** | **P value** |
| PG32:0 | 6.30 | | 1.167 | 10.76 | 3.385 | 0.4046 |
| PG34:1 | 28.37 | | 3.636 | 28.63 | 2.966 | >0.9999 |
| PG36:2 | 11.26 | | 3.105 | 9.81 | 1.361 | >0.9999 |
| PG36:3 | 16.00 | | 2.577 | 19.98 | 0.716 | 0.8526 |

**Supplementary Table 8:** **PUFA supplementation of culture medium alters the lipid membrane profile of *in vitro* differentiated human primary bronchial epithelial cells from subjects with severe asthma.** Matching *in vitro* differentiated PBECs of 3-5 independent subjects with severe asthma were supplemented with arachidonic acid (AA), linoleic acid (LA) or docosahexaenoic acid (DHA) as described in the methods section and the lipid profile analysed by mass spectrometry. Significance was tested against the standard un-supplemented *in vitro* culture using ANOVA and Fisher’s LSD test. b.d.: below detection limit

|  | *in vitro* (n=4) | *in vitro* +AA (n=4) | | *in vitro* +LA (n=4) | | *in vitro* +DHA (n=4) | | *ex vivo* (n=6) |
| --- | --- | --- | --- | --- | --- | --- | --- | --- |
| Phosphatidylcholine | | | | | | | | |
|  | **MEAN±SEM** | **MEAN±SEM** | **P value** | **MEAN±SEM** | **P value** | **MEAN±SEM** | **P value** | **MEAN±SEM** |
| PC30:0 | 3.17±0.295 | 404±0.261 | 0.086 | 3.67±0.262 | 0.887 | 3.67±0.374 | 0.471 | 1.66±0.511 |
| PC32:0 | **5.61±0.671** | **8.51±0.430** | **0.018** | **8.06±0.630** | **0.023** | **7.37±0.546** | **0.019** | 12.45±2.860 |
| PC32:1 | 9.24±0.675 | 9.71±0.662 | >0.999 | 9.01±0.621 | >0.999 | 9.78±0.753 | 0.325 | 2.30±0.661 |
| PC34:1 | 31.11±0.775 | 31.94±1.661 | >0.999 | 29.22±1.256 | 0.476 | 34.66±2.284 | 0.596 | 23.25±0.848 |
| PC34:2 | 9.89±0.177 | 8.51±0.495 | 0.107 | 10.51±0.405 | 0.830 | 8.29±0.536 | 0.169 | 11.82±0.701 |
| PC36:1 | 5.10±0.551 | 4.86±0.448 | >0.999 | 4.03±0.380 | 0.038 | 5.34±0.499 | 0.627 | 7.66±0.660 |
| PC36:2 | **20.80±0.888** | 16.15±0.980 | 0.078 | **15.36±0.759** | 0.022 | 17.55±1.064 | 0.314 | 12.06±1.141 |
| PC36:3 | **3.37±0.089** | 2.95±0.135 | 0.109 | **6.84±0.527** | 0.027 | **2.26±0.069** | **0.007** | 6.76±0.578 |
| PC36:4 | **0.458±0.085** | **1.71±0.264** | **0.036** | 1.41±0.195 | 0.074 | 0.49±0.025 | >0.999 | 5.65±0.550 |
| PC38:2 | **1.87±0.209** | **1.28±0.141** | **0.043** | 1.38±0.285 | 0.570 | 1.87±0.275 | >0.999 | 0.88±0.148 |
| PC38:3 | **2.53±0.210** | 2.24±0.202 | 0.164 | 2.60±0.252 | >0.999 | **1.71±0.135** | **0.021** | 3.32±0.472 |
| PC38:4 | **0.95±0.011** | 2.06±0.242 | 0.070 | **2.44±0.217** | **0.025** | **0.68±0.018** | **0.003** | 6.53±0.695 |
| PC38:6 | **0.07±0.013** | 0.16±0.044 | 0.361 | 0.12±0.024 | 0.194 | **1.01±0.134** | **0.025** | 1.66±0.082 |
| PC40:6 | 0.09±0.019 | 0.26±0.069 | 0.359 | 0.16±0.032 | 0.526 | 0.34±0.054 | 0.101 | 1.10±0.103 |
| Others |  |  |  |  |  |  |  |  |
| PC32a:0 | 1.11±0.141 | 0.95±0.119 | 0.436 | 0.88±0.0992 | 0.122 | 0.89±0.157 | 0.197 | 0.26±0.029 |
| PC32a:1 | 0.91±0.159 | 1.22±0.165 | 0.187 | 1.13±0.086 | 0.372 | 0.82±0.148 | 0.741 | 0.86±0.108 |
| PC34a:2 | 2.23±0.254 | 2.25±0.322 | >0.999 | 1.99±0.204 | >0.999 | 1.95±0.261 | >0.999 | 1.12±0.050 |
| PC34a:1 | 1.51±0.151 | 1.23±0.158 | 0.599 | 1.23±0.158 | 0.090 | 1.35±0.146 | 0.869 | 0.68±0.082 |
|  | ***in vitro* (n=5)** | ***in vitro* +AA (n=5)** | | ***in vitro* +LA (n=4)** | | ***in vitro* +DHA (n=3)** | | ***ex vivo* (n=5)** |
| Phosphoinositol | | | | | | | | |
|  | **MEAN±SEM** | **MEAN±SEM** | **P value** | **MEAN±SEM** | **P value** | **MEAN±SEM** | **P value** | **MEAN±SEM** |
| PI32:0 | 0.68±0.081 | 0.61±0.205 | >0.999 | 0.77±0.340 | >0.999 | 0.91±0.255 | >0.999 | 1.38±0.2985 |
| PI32:1 | 1.69±0.079 | 2.04±0.286 | 0.880 | 1.61±0.294 | >0.999 | 1.76±0.297 | >0.999 | 0.49±0.0301 |
| PI34:1 | 11.02±1.167 | 11.42±1.879 | >0.999 | 9.77±1.780 | 0.559 | 14.08±2.585 | 0.523 | 11.19±0.717 |
| PI34:2 | 4.08±0.297 | 3.39±0.130 | 0.486 | 3.15±0.293 | 0.584 | 3.84±0.072 | >0.999 | 4.08±0.532 |
| PI36:1 | 12.18±2.027 | 10.37±2.046 | 0.662 | 10.14±1.451 | 0.249 | 14.07±1.268 | 0.651 | 11.25±0.814 |
| PI36:2 | **18.07±1.604** | **11.48±0.803** | **0.019** | 12.65±0.594 | 0.061 | 15.59±1.529 | 0.458 | 11.14±0.826 |
| PI36:3 | 5.49±0.378 | 4.02±0.246 | 0.250 | 6.87±0.772 | 0.520 | 5.25±0.358 | 0.391 | 4.07±0.691 |
| PI36:4 | **0.91±0.170** | **3.75±0.523** | **0.023** | **1.22±0.164** | **0.013** | 1.12±0.341 | >0.999 | 6.19±0.523 |
| PI38:3 | 35.03±3.268 | 20.71±2.254 | 0.225 | 35.24±3.053 | >0.999 | 31.61±2.313 | 0.403 | 12.94±2.186 |
| PI38:4 | 10.86±2.194 | 32.22±6.942 | 0.094 | 18.60±4.237 | 0.200 | 11.78±2.979 | >0.999 | 37.27±3.273 |

**Supplementary Table 8 (continued):** **PUFA supplementation of culture medium alters the lipid membrane profile of *in vitro* differentiated human primary bronchial epithelial cells from subjects with severe asthma.** Matching *in vitro* differentiated PBECs of 3-5 independent subjects with severe asthma were supplemented with arachidonic acid (AA), linoleic acid (LA) or docosahexaenoic acid (DHA) as described in the methods section and the lipid profile analysed by mass spectrometry. Significance was tested against the standard un-supplemented *in vitro* culture using ANOVA and Fisher’s LSD test. b.d.: below detection limit

|  | *in vitro* (n=5) | | | | *in vitro* +AA (n=5) | | | | | *in vitro* +LA (n=4) | | | | *in vitro* +DHA (n=3) | | | | *ex vivo* (n=5) | |  |  |
| --- | --- | --- | --- | --- | --- | --- | --- | --- | --- | --- | --- | --- | --- | --- | --- | --- | --- | --- | --- | --- | --- |
| phosphatidylethanolamine | | | | | | | | | | | | | | | | | | | | |  |
|  | **MEAN±SEM** | | **MEAN±SEM** | | | | **P value** | | **MEAN±SEM** | | **P value** | | **MEAN±SEM** | | **P value** | | **MEAN±SEM** | |  |  |  |
| PE32:1 | 1.88±0.083 | | 1.64±0.082 | | | | 0.760 | | 1.57±0.150 | | 0.732 | | 1.74±0.111 | | >0.999 | | 0.21±0.051 | |  |  |  |
| PE34:1 | 14.77±0.409 | | 13.21±0.695 | | | | 0.721 | | 13.51±0.581 | | 0.449 | | 15.62±1.566 | | >0.999 | | 7.70±0.493 | |  |  |  |
| PE34:2 | **5.75±0.118** | | 4.55±0.180 | | | | 0.080 | | 5.70±0.383 | | >0.999 | | **4.09±0.216** | | **0.010** | | 2.83±0.187 | |  |  |  |
| PE36:1 | 15.90±2.375 | | 20.30±0.486 | | | | 0.504 | | 18.52±0.785 | | 0.892 | | 18.41±1.136 | | 0.674 | | 21.78±0.865 | |  |  |  |
| PE36:2 | **33.03±0.974** | | **23.36±1.899** | | | | 0.033 | | 27.22±1.021 | | 0.081 | | **22.36±0.377** | | 0.013 | | 12.09±0.716 | |  |  |  |
| PE36:3 | 4.90±0.269 | | 4.19±0.097 | | | | 0.276 | | **7.34±0.322** | | **0.021** | | 2.84±0.482 | | 0.134 | | 4.47±0.365 | |  |  |  |
| PE38:2 | 3.80±1.275 | | 7.35±0.984 | | | | 0.241 | | 4.80±0.650 | | >0.999 | | 3.87±0.122 | | >0.999 | | 4.59±0.230 | |  |  |  |
| PE38:3 | 6.56±0.703 | | 6.76±0.857 | | | | >0.999 | | 9.07±0.754 | | 0.696 | | 9.57±1.153 | | 0.451 | | 12.32±0.786 | |  |  |  |
| PE38:4 | 2.12±1.243 | | 4.04±1.393 | | | | >0.999 | | 3.49±0.672 | | 0.472 | | 0.00±0.000 | | 0.749 | | 3.22±0.902 | |  |  |  |
| PE38:5 | b.d. | | 0.45±0.178 | | | |  | | b.d. | |  | | b.d. | |  | | 1.92±0.345 | |  |  |  |
| PE38:6 | **0.82±0.151** | | 0.46±0.189 | | | | 0.832 | | **0.04±0.043** | | **0.044** | | **3.75±0.580** | | **0.028** | | 3.62±0.481 | |  |  |  |
| PE40:3 | 0.92±0.362 | | 1.47±0.057 | | | | 0.904 | | 1.40±0.240 | | >0.999 | | 2.01±0.372 | | 0.745 | | 2.80±0.400 | |  |  |  |
| PE40:4 | 1.05±0.291 | | 1.21±0.406 | | | | >0.999 | | 1.47±0.386 | | 0.696 | | 3.45±0.619 | | 0.109 | | 1.65±0.303 | |  |  |  |
| PE40:5 | 1.89±1.776 | | 0.24±0.159 | | | | >0.999 | | 0.54±0.212 | | >0.999 | | 0.08±0.078 | | >0.999 | | 1.39±0.347 | |  |  |  |
| PE40:6 | 3.42±1.966 | | 1.11±0.239 | | | | >0.999 | | 0.29±0.087 | | 0.848 | | 2.25±0.472 | | >0.999 | | 2.07±0.408 | |  |  |  |
| PE40:7 | 2.76±0.950 | | 7.74±2.478 | | | | 0.402 | | 3.95±0.600 | | 0.189 | | 6.88±0.919 | | 0.236 | | 12.35±0.859 | |  |  |  |
|  | ***in vitro*** | | ***in vitro*+AA** | | | | | | ***in vitro*+LA** | | | | | ***in vitro*+DHA** | | | | ***ex vivo*** | | | |
| phosphatidylserine | | | | | | | | | | | | | | | | | | | | |  |
|  | **MEAN±SEM** | | | **MEAN±SEM** | | | **P value** | | **MEAN±SEM** | | **P value** | | | **MEAN±SEM** | | **P value** | | **MEAN±SEM** | |  |  |
| PS36:1 | 65.58±2.770 | | | 68.17±2.078 | | | >0.999 | | 64.01±1.572 | | >0.999 | | | 72.64±1.279 | | 0.116 | | 65.71±2.420 | |  |  |
| PS38:3 | **5.69±0.614** | | | 5.48±0.649 | | | >0.999 | | 10.76±1.313 | | 0.136 | | | **3.09±0.338** | | **0.015** | | 11.29±1.679 | |  |  |
| PS40:4 | **1.65±0.298** | | | 4.10±1.487 | | | 0.537 | | 2.00±0.480 | | >0.999 | | | **0.78±0.198** | | **0.025** | | 4.51±0.792 | |  |  |
| PS40:5 | 0.87±0.200 | | | 1.27±0.252 | | | 0.821 | | 0.85±0.267 | | >0.999 | | | 0.98±0.296 | | >0.999 | | 7.13±1.114 | |  |  |
| PS40a:8 | 2.41±0.297 | | | 2.66±0.239 | | | >0.999 | | 2.73±0.253 | | >0.999 | | | 3.19±0.217 | | 0.089 | | 2.30±0.113 | |  |  |
| PS40a:9 | 12.59±1.780 | | | 9.23±0.445 | | | 0.465 | | 9.84±1.142 | | 0.279 | | | 9.90±1.129 | | 0.129 | | 4.52±0.386 | |  |  |
| PS42a:8 | **1.58±0.291** | | | 1.93±0.456 | | | >0.999 | | 1.94±0.330 | | 0.279 | | | **2.64±0.297** | | **0.011** | | 1.09±0.144 | |  |  |
| PS42a:9 | 4.95±0.438 | | | 3.84±0.337 | | | 0.729 | | 4.40±0.239 | | >0.999 | | | 3.99±0.498 | | >0.999 | | 2.75±0.170 | |  |  |
| PS42a:10 | 4.69±0.698 | | | 3.33±0.642 | | | 0.906 | | 3.49±0.308 | | 0.225 | | | 2.80±0.334 | | 0.101 | | 0.70±0.139 | |  |  |
|  | ***in vitro*** | | | ***in vitro*+AA** | | | | | ***in vitro*+LA** | | | | | ***in vitro*+DHA** | | | | ***ex vivo*** | |  |  |
| Phosphatidylglycerol | | | | | | | | | | | | | | | | | | | |  |  |
|  | | **MEAN±SEM** | | | | **MEAN±SEM** | | **P value** | | **MEAN±SEM** | | **P value** | | **MEAN±SEM** | | **P value** | | **MEAN**±SEM | |  |  |
| PG32:0 | | 10.76±3.358 | | | | 11.28±2.716 | | >0.999 | | 5.32±1.935 | | >0.999 | | 13.08±1.665 | | >0.999 | | 6.30±1.167 | |  |  |
| PG34:1 | | 28.63±2.966 | | | | 21.56±1.544 | | >0.999 | | 27.27±6.134 | | >0.999 | | 29.46±3.557 | | >0.999 | | 28.37±3.636 | |  |  |
| PG36:2 | | **19.98±0.716** | | | | 16.09±1.544 | | 0.678 | | 14.55±4.039 | | >0.999 | | **7.465±2.735** | | **0.045** | | 16.00±2.577 | |  |  |
| PG36:3 | | 9.81±1.361 | | | | 16.02±5.106 | | >0.999 | | 15.71±3.924 | | >0.999 | | 10.73±1.756 | | >0.999 | | 11.26±3.105 | |  |  |
