# Supplementary material for: Membrane lipid composition of bronchial epithelial cells influences antiviral responses during rhinovirus infection
Source: Tissue Barriers. 2024 Jan 5;12(4):2300580. doi: 10.1080/21688370.2023.2300580 (PMC11583602; doi:10.1080/21688370.2023.2300580)
Supplement: Panchal et al supplementary figures.pdf [file KTIB_A_2300580_SM9030.pdf]

Supplementary Figure 1: Glycerophospholipids are classed into groups according to their head group. Fatty acids are bound to glycerol at the sn-1 and sn-2 position, with the sn-1 position taken preferentially by saturated fatty acids while the sn-2 position can hold saturated, mono-unsaturated or poly-unsaturated fatty acids. To simplify the nomenclature of fatty acids, they are commonly referred to by the number of carbon atoms followed by the number of double bonds after a colon. For unsaturated fatty acids, the position of the double bonds can be noted in the form n-x, where x indicates the position of the carbon atom with the first double bond counted from the methyl end of the carbon chain. For example, arachidonic acid is expressed as 20:4n-6, as it has 20 carbon atoms, 4 double bonds and the first double bond is on the 6th carbon atom.

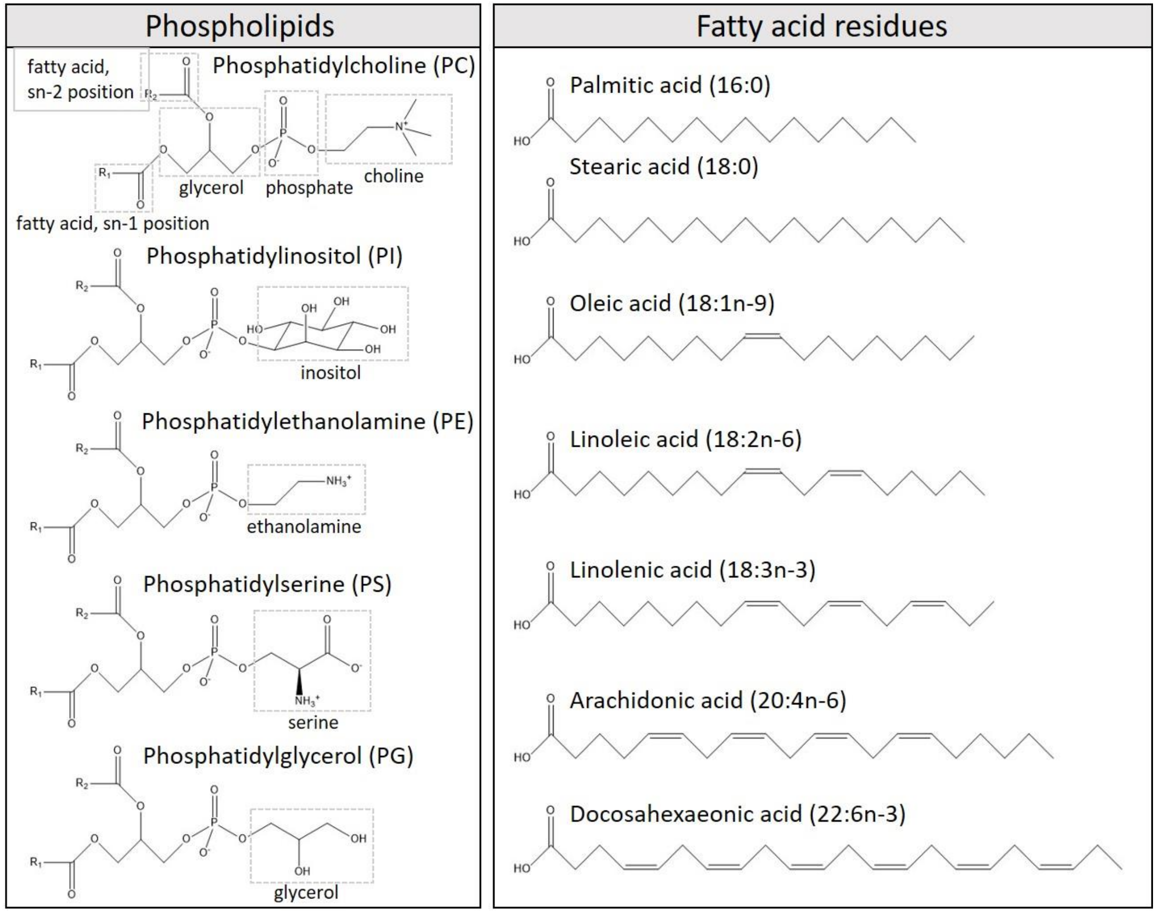

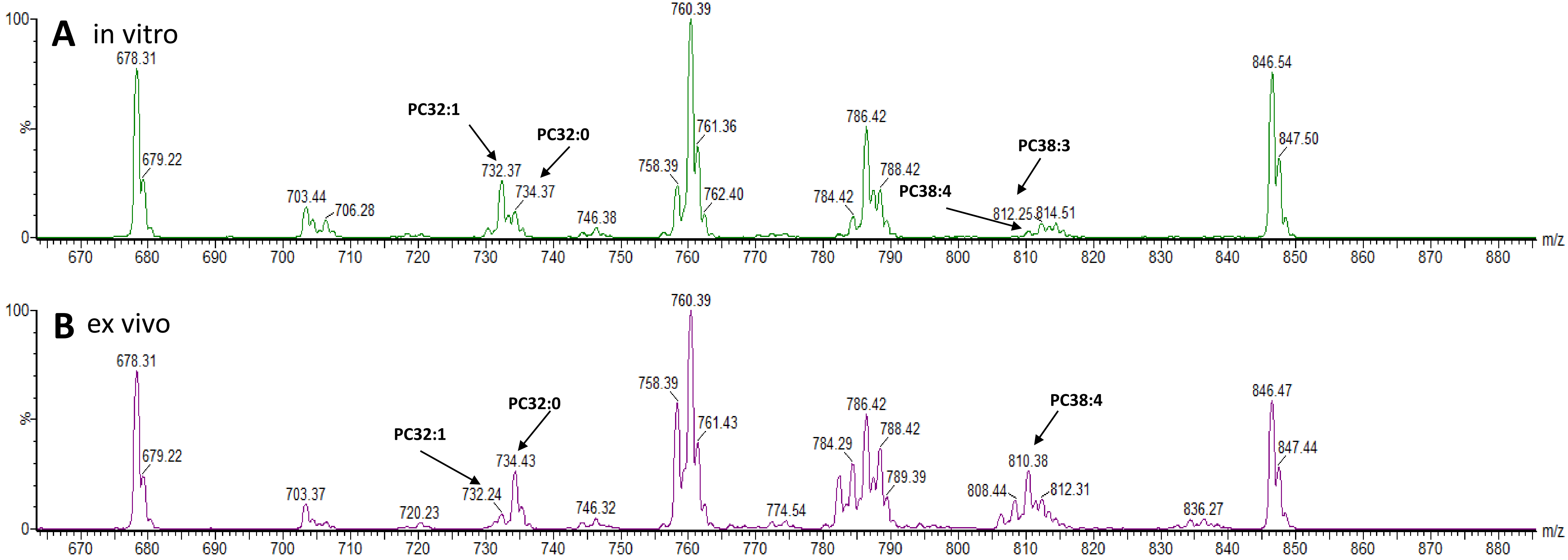

Supplementary Figure 2: Phosphatidylcholine (PC) composition of the lipid membrane of *ex vivo* bronchial brushings and *in vitro* differentiated human primary bronchial epithelial cells (PBECs). Representative mass spectra of matching *ex vivo* (A) and *in vitro* (B) samples of a total n=5 individual subjects.

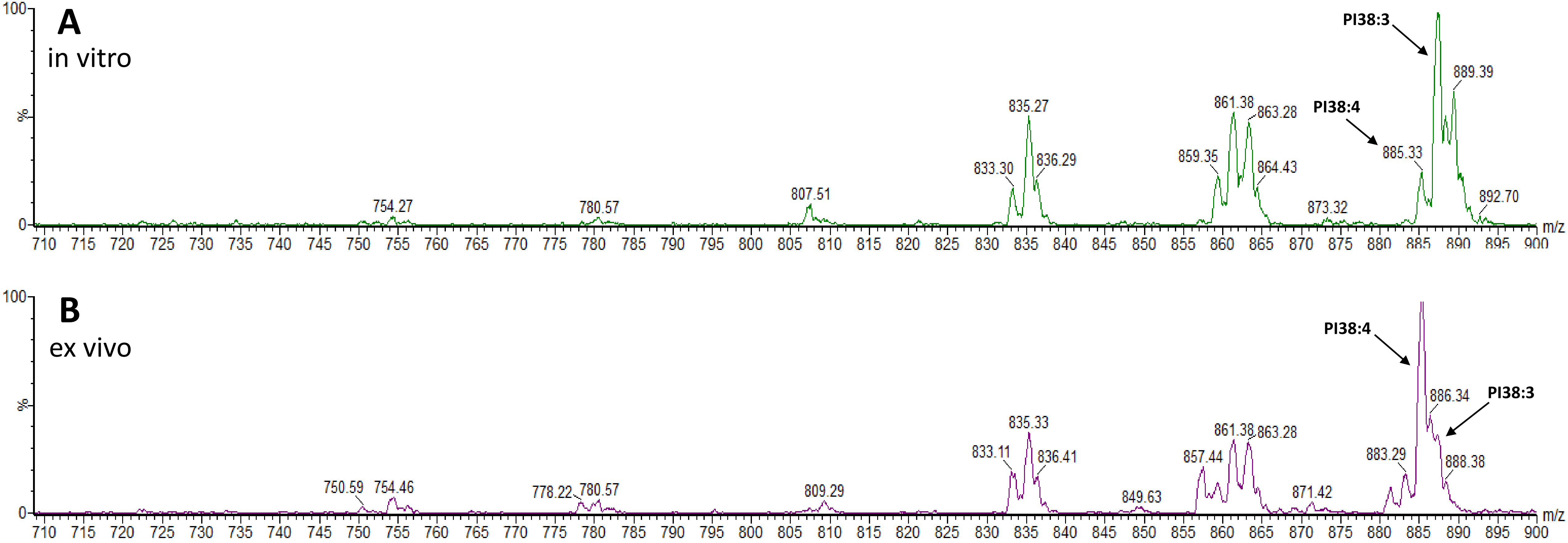

Supplementary Figure 3: Phosphatidylinositol (PI) composition of the lipid membrane of *ex vivo* bronchial brushings and *in vitro* differentiated human primary bronchial epithelial cells (PBECs). Representative mass spectra of matching *ex vivo* and *in vitro* samples of a total n=5 individual subjects.

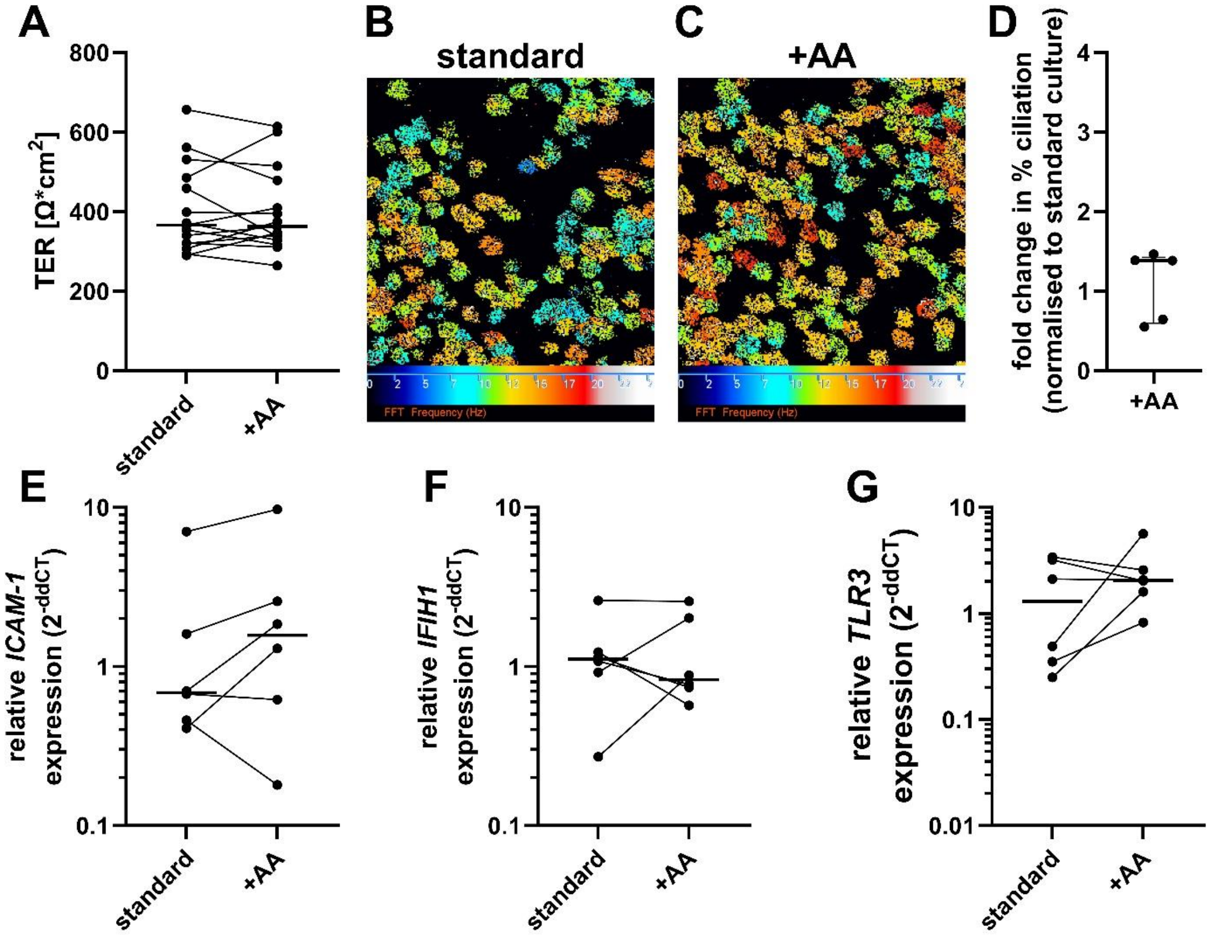

Supplemental Figure 4: AA supplementation does not affect the differentiation status nor the expression of key genes involved in RV16 cell entry and recognition in bronchial epithelial cells derived from non-asthmatic donors. PBECs from non-asthmatic donors were differentiated at the air-liquid interface for 21 days and supplemented with AA as described in the methods. A: Transepithelial electrical resistance (TER) of differentiated PBECs following AA supplementation. B-D: The surface area of differentiated cultures covered by moving cilia was determined by high speed video microscopy in standard (B) and AA-supplemented cultures (C). From 5 areas spanning across the well the average area covered by moving cilia was determined as % ciliation and expressed as fold change normalised to the unsupplemented standard culture conditions (E). E-G: Expression of *ICAM-1* (E), *IFIH1* (F) and *TLR3* (G) was determined by qPCR in differentiated PBECs with and without AA supplementation.

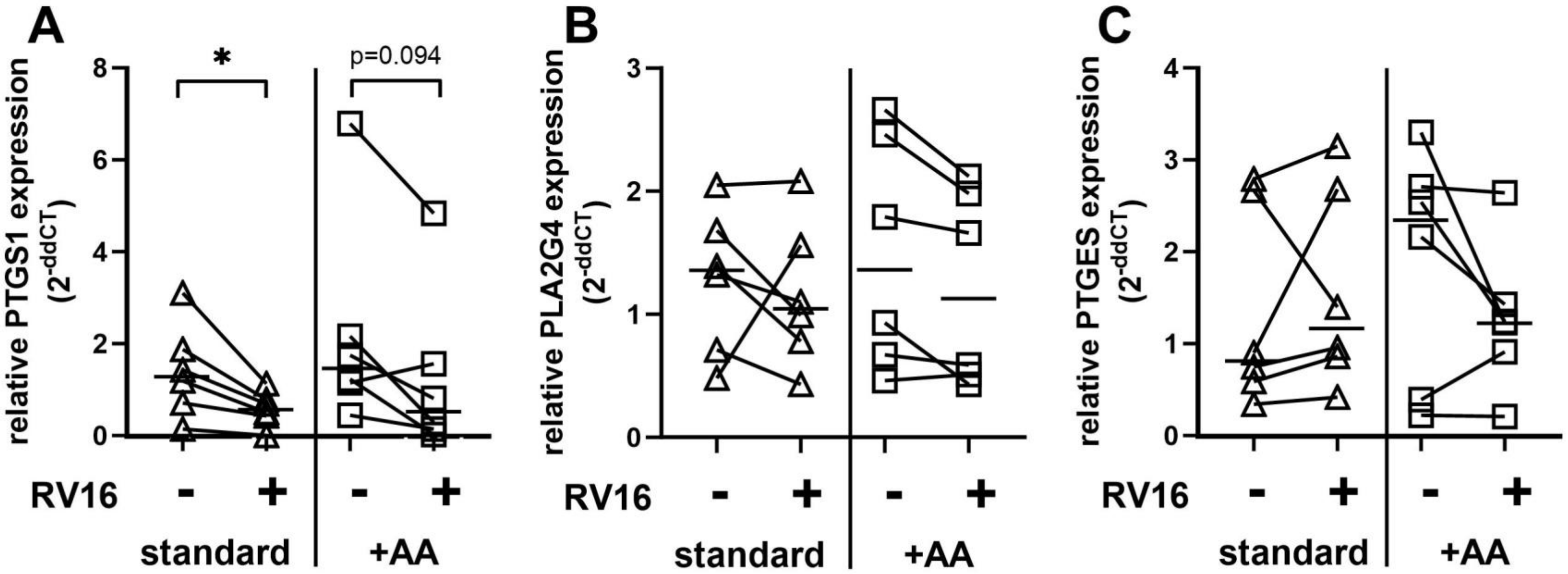

Supplemental Figure 5: Expression of other rate-limiting enzymes in the PGE_2 synthesis cascade is not changed. Expression of PTGS1 (A), PLA2G4 (B) and PTGES (C) was determined by qPCR in differentiated PBECs with and without AA supplementation following 24h of RV16 infection. Non-asthmatic subjects; n=6. *: $p \leq 0.05$, non-parametric Wilcoxon test for paired samples.

Supplemental Figure 6: The lipid membrane profile of PBECs derived from subjects without or with severe asthma are similar. The profile of phoshatidylinositol species was determined by mass spectrometry. A: Comparison of *ex vivo* bronchial brushings from healthy, non-asthmatic subjects (n=5) and subjects with severe asthma (n=6). B: Comparison of *ex vivo* bronchial bushings and *in vitro* differentiated PBECs using standard culture conditions (n=4). MEAN +/-SEM. *: p≤0.05, **: p≤0.01 2-way ANOVA with Bonferroni multiple comparison test. C: comparison of differentiated PBECs using standard culture conditions and supplemented with arachidonic acid (AA), linoleic acid (LA) or docosahexaenoic acid (DHA) (n=4). For comparison, the PI profile of *ex vivo* bronchial brushings are shown (n=6).

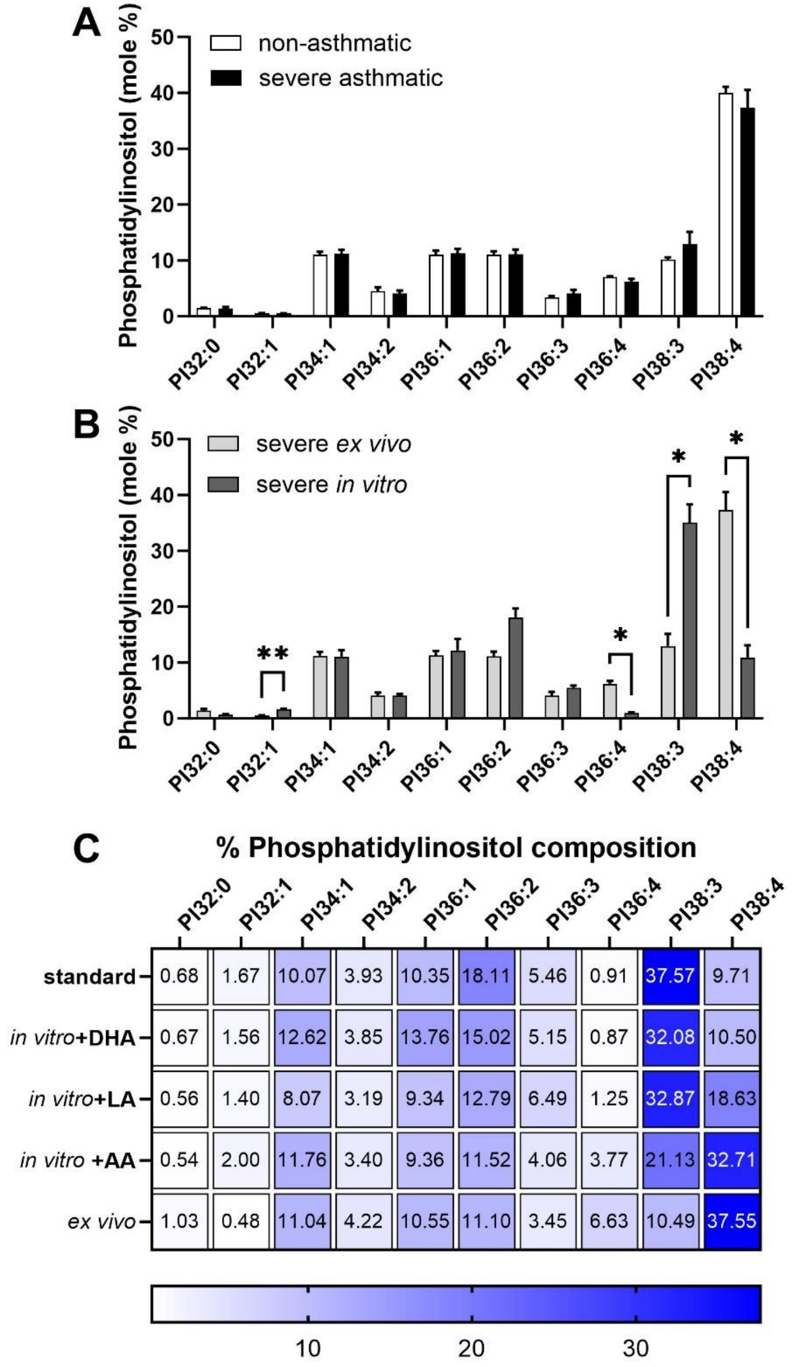

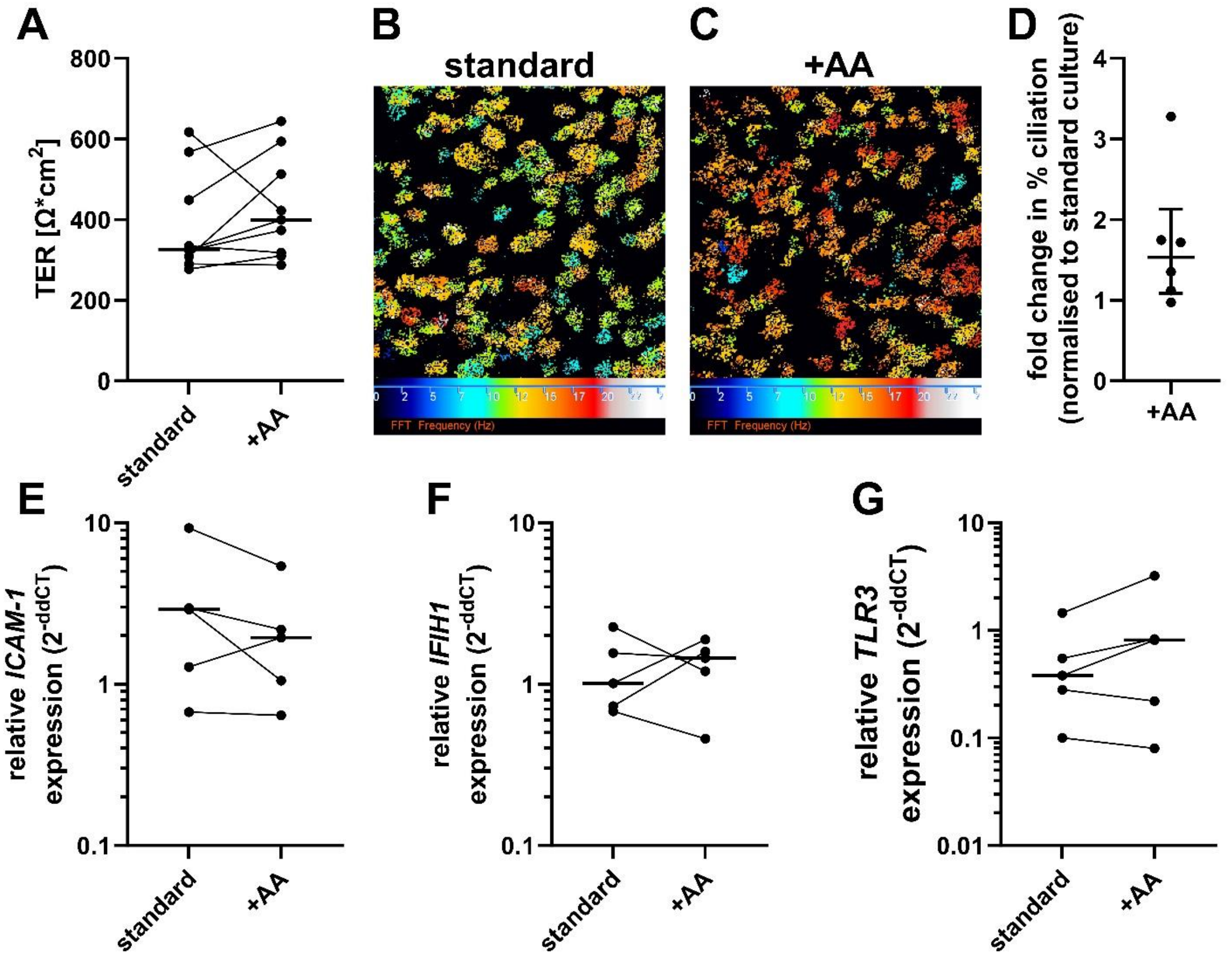

Supplemental Figure 7: AA supplementation does not affect the differentiation status nor the expression of key genes involved in RV16 cell entry and recognition in bronchial epithelial cells derived from patients with severe asthma. PBECs from patients with severe asthma were differentiated at the air-liquid interface for 21 days and supplemented with AA as described in the methods. A: Transepithelial electrical resistance (TER) of differentiated PBECs following AA supplementation. B-D: The surface area of differentiated cultures covered by moving cilia was determined by high speed video microscopy in standard (B) and AA-supplemented cultures (C). From 5 areas spanning across the well the average area covered by moving cilia was determined as % ciliation and expressed as fold change normalised to the unsupplemented standard culture conditions (D). E-G: Expression of *ICAM-1* (E), *IFIH1* (F) and *TLR3* (G) was determined by qPCR in differentiated PBECs with and without AA supplementation.

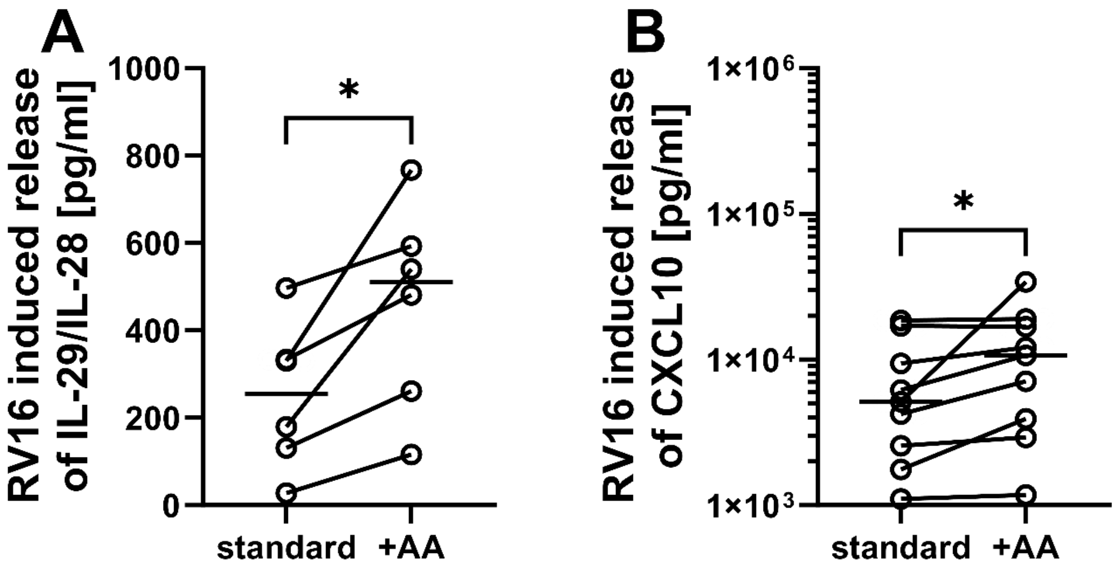

Supplemental Figure 8: AA supplementation of *in vitro* differentiated human PBECs derived from subjects with severe asthma increases the release of IL-29/IL-28 and IP-10 during rhinovirus infection. Cultures were infected with human rhinovirus 16 (RV16) for 24hrs before supernatants were taken. Standard un-supplemented cultures were used as control. Release of IFNλ (IL29/IL28) (A) and CXCL10 (IP-10) (B) was analysed by ELISA. A: n=6; B: n=9; *: $p \leq 0.05$ non-parametric Wilcoxon test.
